# Fault-Tolerant Private Information Retrieval via Threshold Distributed Point Functions

**DOI:** 10.3390/e28090945

**Published:** 2026-08-23

**Authors:** Dazeng Yuan, Xiheng Liu, Bin Liu

**Affiliations:** 1 Department of Basic Medicine, Dazhou Vocational College of Chinese Medicine, Dazhou 635000, China; yuan_dz34@163.com; 2 School of Information Science and Technology, Southwest Jiaotong University, Chengdu 610032, China; xihengliu7@gmail.com

**Keywords:** private information retrieval, function secret sharing, distributed point function, secure multi-party computation

## Abstract

Multi-server private information retrieval (PIR) based on function secret sharing (FSS) has emerged as a prominent paradigm for achieving sublinear communication. However, standard FSS constructions require full server participation, making them highly vulnerable to single-node fail-stop faults. Existing fault-tolerant schemes mitigate this but inevitably inflate the response overhead to scale with the database size *N* (e.g., $O(\sqrt{N})$). To overcome this limitation, we propose a fault-tolerant PIR (FT-PIR) protocol based on a newly designed (t,p)-threshold distributed point function (FT-DPF). By introducing a hierarchical recursive patching mechanism, our scheme transforms rigid all-party evaluations into flexible *t*-out-of-*p* reconstructions. This architecture completely decouples the response communication from *N* and ensures efficient client-side reconstruction via lightweight XOR aggregations. Formal analysis proves that our stateless protocol guarantees (t−1)-computational privacy under the semi-honest model. Theoretical analysis demonstrates that the proposed FT-PIR achieves a response complexity bounded by $O(F_{\mathrm{maxlevel}}\left(t,p\right))$. Comprehensive experimental evaluations confirm that our implementation significantly reduces practical communication and computation overheads, outperforming the state-of-the-art scheme.

## 1. Introduction

Private information retrieval (PIR) is a fundamental cryptographic primitive that enables a client to retrieve specific records from a public database containing *N* items without revealing the query index [1]. As a cornerstone for privacy-preserving data access, PIR has seen widespread adoption in private contact discovery [2], private web search [3], anonymity networks [4], and encrypted search systems [5]. Historically, PIR research has bifurcated into two primary architectures based on the underlying trust model: single-server and multi-server PIR.

Single-server PIR operates without non-collusion assumptions and is predominantly driven by two technical paradigms: homomorphic encryption (HE) and hint-based preprocessing. However, both paradigms encounter inherent mechanistic bottlenecks. In HE-based constructions, schemes employing packing techniques to optimize bandwidth [6] suffer from prohibitive homomorphic computational overheads. Even with homomorphic Thorp shuffles to minimize circuit depth [7], the high constant-factor costs of HE evaluations remain a significant barrier. To accelerate online performance, recent schemes introduced preprocessing mechanisms [8] to achieve sublinear query times, albeit by imposing substantial state storage overhead on the client. Alternatively, a parallel route leverages hint-based preprocessing [9,10,11,12]. While these schemes utilize lightweight symmetric primitives to yield attractive time–space trade-offs, they mandatorily require exhaustive offline database scans. Furthermore, their hint mechanisms face periodic and heavy reconstruction burdens due to limited query budgets, restricting their practical viability in high-frequency scenarios.

To circumvent the performance bottlenecks of single-server models, multi-server PIR achieves highly efficient retrieval by distributing trust across *p* non-colluding servers. Under this architecture, information-theoretic (IT) constructions based on algebraic coding have been extensively studied [13]. These schemes utilize polynomials and erasure codes to establish a communication efficiency of $O(N^{1/3})$ [14] and optimal robustness bounds [15], ultimately achieving sub-polynomial communication via matching vector codes [16]. Despite these theoretical milestones, IT-PIR typically involves complex finite-field operations that concretely diminish their practical bandwidth advantages. Although recent advancements like multi-server preprocessing [17] attempt to amortize online computation, they inevitably force the client to maintain persistent states, failing to simultaneously balance sub-polynomial communication, stateless operation, and efficient fault tolerance.

Parallel to IT-PIR, the function secret sharing (FSS) paradigm has emerged as an efficient approach for constructing lightweight multi-server PIR [18,19]. Specifically, its core instantiation for point queries—the distributed point function (DPF)—enables clients to compress query key overhead to O(logN). However, scaling standard DPFs from a two-server to a *p*-server environment (p≥3) initially introduced severe scalability issues, with request bandwidth exhibiting exponential growth ($O(2^{p}\sqrt{N})$) relative to the number of servers [18]. While recent combinatorial design optimizations have successfully compressed this multi-party key size to $O(p^{3}\sqrt{N})$ [20], a far more critical vulnerability persists: standard FSS-based PIR protocols are exceptionally fragile as they require all *p* servers to remain online during the evaluation and reconstruction phases. While recent state-of-the-art works have introduced fault-tolerant DPF mechanisms to enhance system robustness [21], such approaches inevitably inflate the response overhead to $O(\sqrt{N})$, essentially tying the downlink cost to the database size.

To overcome this limitation, we propose a novel fault-tolerant PIR scheme (FT-PIR) grounded in a newly designed fault-tolerant DPF (FT-DPF). By leveraging a recursive patching mechanism, our scheme successfully generalizes a base (2,p)-threshold DPF into a (t,p)-threshold setting. This architecture enables the client to reconstruct the target data using responses from any *t* surviving servers. Consequently, as compared in Table 1, our protocol achieves a robust and lightweight retrieval process that effectively decouples the response complexity from *N*.

### 1.1. Our Contributions

The main contributions of this paper are summarized as follows:

- A Novel (t,p)-Fault-Tolerant DPF Scheme: We formally define and construct a generalized (t,p)-fault-tolerant distributed point function (FT-DPF) through a hierarchical, bottom-up design. Specifically, we first optimize the key length of the state-of-the-art multi-party DPF [20]. Building upon this optimized DPF, we construct a highly efficient base (2,p) DPF scheme. Finally, we introduce a recursive patching mechanism to extend this base construction into a generalized (t,p)-threshold setting. This allows any *t* surviving servers to correctly reconstruct the evaluation, thereby tolerating up to p−t fail-stop failures.
- FT-PIR Protocol with Database-Size-Independent Response Overhead: Built upon our generalized FT-DPF, we propose a stateless, fault-tolerant PIR protocol. Unlike Shamir-based schemes burdened by an $O(\sqrt{N})$ response overhead, our architecture completely decouples response communication from *N*, bounding the complexity to $O(F_{\mathrm{maxlevel}}\left(t,p\right))$. Given that practical non-colluding deployments treat *p* as a small constant relative to large-scale datasets (N≫p≥t), this design achieves flexible scalability for large-scale datasets. Furthermore, the client-side reconstruction relies purely on straightforward XOR aggregations, avoiding the heavy algebraic interpolations typical of traditional robust schemes.
- Formal Security and Performance Evaluation: We prove (t−1)-computational privacy under the semi-honest model. Theoretical and empirical results demonstrate that our protocol decouples response overhead from the database size and achieves sublinear queries, significantly outperforming the state-of-the-art [21].

### 1.2. Organization

The remainder of this paper is organized as follows. Section 2 introduces the necessary cryptographic preliminaries. Section 3 formalizes the system model and definitions. Building upon this foundation, Section 4 details the core technical contribution: the hierarchical construction of the generalized threshold DPF. Section 5 then deploys this primitive to construct the fault-tolerant PIR protocol. Section 6 formalizes the composite security paradigm and provides theoretical proofs for query privacy and fault tolerance. Section 7 presents a comprehensive efficiency evaluation, encompassing both theoretical analysis and experimental comparisons. Section 8 reviews the existing literature on single-server and multi-server PIR architectures. Finally, Section 9 concludes the paper and outlines directions for future work.

## 2. Preliminaries

In this section, we introduce some essential preliminary concepts such as DPF [18,20] and replicated secret sharing [22]. Table 2 lists the key notations used in this paper.

### 2.1. Distributed Point Function

A distributed point function (DPF) [18,19] is a cryptographic primitive that enables a client to secretly share a point function across multiple servers. Let (G,+) be an Abelian group. A point function $f_{\alpha ,\beta }:{\left\{0,1\right\}}^{n}\to G$ is defined such that $f_{\alpha ,\beta }\left(x\right)=\beta$ if x=α, and 0 otherwise, where $\alpha \in {\left\{0,1\right\}}^{n}$ is the target input and β∈G∖{0} is the non-zero output value. In a *p*-party DPF, the client splits $f_{\alpha ,\beta }$ into *p* distinct keys $k_{1},\ldots ,k_{p}$. This splitting guarantees that any strict subset of the keys reveals no information about the secret parameters (α,β), yet the aggregated evaluations of all keys at a given point *x* correctly reconstruct $f_{\alpha ,\beta }\left(x\right)$. Conceptually, these keys act as secret shares of a vector of length $N=2^{n}$, where all elements are zero except for the index corresponding to α. Formally, a DPF scheme is defined as follows:

**Definition 1** 
(Distributed Point Function [18])**.** *A p-party DPF scheme over the domain ${\left\{0,1\right\}}^{n}$ and an Abelian group (G,+) comprises two probabilistic polynomial-time (PPT) algorithms (Gen,Eval) satisfying the following properties:*

- *Syntax:*
  – *$\left(k_{1},\ldots ,k_{p}\right)\leftarrow \mathrm{Gen}\left(1^{\lambda },\alpha ,\beta \right)$: Takes as input a security parameter λ, a target index $\alpha \in {\left\{0,1\right\}}^{n}$, and a payload β∈G. It outputs a p-tuple of keys $\left(k_{1},\ldots ,k_{p}\right)$.*
  – *$y_{i}\leftarrow \mathrm{Eval}\left(i,k_{i},x\right)$: Takes as input a party index i∈[p], the corresponding key $k_{i}$, and an evaluation point $x\in {\left\{0,1\right\}}^{n}$. It outputs a group element $y_{i}\in G$.*
- *Correctness: For any $\alpha \in {\left\{0,1\right\}}^{n}$, β∈G, and any $x\in {\left\{0,1\right\}}^{n}$, if $\left(k_{1},\ldots ,k_{p}\right)\leftarrow \mathrm{Gen}\left(1^{\lambda },\alpha ,\beta \right)$, then it holds with probability* 1 *that $\sum_{i=1}^{p}\mathrm{Eval}\left(i,k_{i},x\right)=f_{\alpha ,\beta }\left(x\right)$.*
- *Privacy: For any strict subset of indices I⊂[p] (i.e., |I|<p), the joint distribution of the keys ${\left\{k_{i}\right\}}_{i\in I}$ generated by $\mathrm{Gen}(1^{\lambda },\alpha ,\beta )$ is computationally indistinguishable from a distribution of keys simulated without the knowledge of (α,β).*

### 2.2. Multi-Party DPF from Special Combinatorial Design

**Figure 1:**
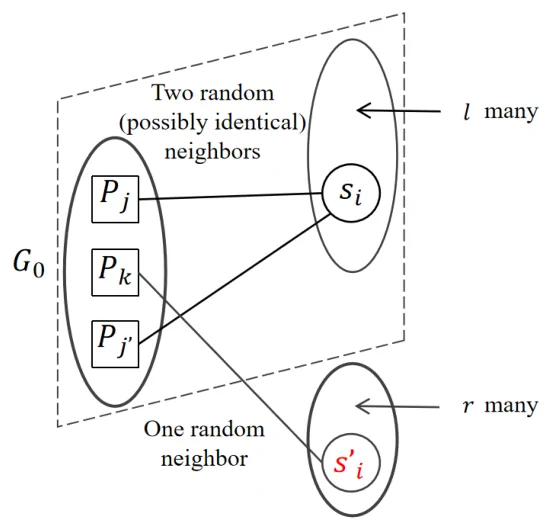
The randomized construction: the highlighted part constitutes $G_{0}$, which contains *l* vertices $s_{1},\ldots ,s_{l}$, whereas $G_{1}$ consists of the entire graph. The two random neighbors of each $s_{i}$ are sampled with replacement (i.e., they could be identical). In addition, $G_{1}$ additionally plant *r* more vertices $s_{1}^{\prime },\ldots ,s_{r}^{\prime }$, each of which has only one random neighbor.

While prior multi-party DPF constructions [18,19] implicitly rely on a *deterministic* combinatorial design that inherently incurs an $O(2^{p})$ key size, the approach presented here circumvents this exponential lower bound by employing a *randomized* combinatorial design [20]. Both approaches can be unified under the abstract mathematical framework of a *special combinatorial design* on bipartite graphs as shown in Figure 1. By leveraging the randomized variant, the key size is drastically reduced to be polynomial in the number of parties *p*.

**Definition 2** 
(Special Combinatorial Design [20])**.** *Let λ,p∈N and ℓ be a polynomial in λ,p. A special combinatorial design consists of two (possibly random) bipartite graphs $G_{0}$ and $G_{1}$, each with p vertices on the left-hand side and ℓ=ℓ(λ,p) vertices on the right-hand side, satisfying:*

*1.*  *(1−η)-Correctness: The probability that each right vertex in $G_{0}$ has an even degree is at least 1−η.*
*2.*  *(1−ζ)-Pseudorandomness: With probability ≥1−ζ, for each left vertex in $G_{1}$, there exists a right vertex to which it is exclusively connected.*
*3.*  *ϵ-Privacy: The statistical distance between the subgraphs obtained by removing the i-th (for any i∈[p]) left vertices from $G_{0}$ and $G_{1}$ is at most ϵ.*

*Here η, ζ, and ϵ are functions of the security parameter λ and p, representing the correctness error, pseudorandomness error, and privacy leakage bound, respectively. In this framework, ℓ corresponds to the number of right vertices in the combinatorial design, representing the number of PRG seeds sampled per row, which directly dictates the key size overhead.*

**Figure 2:**
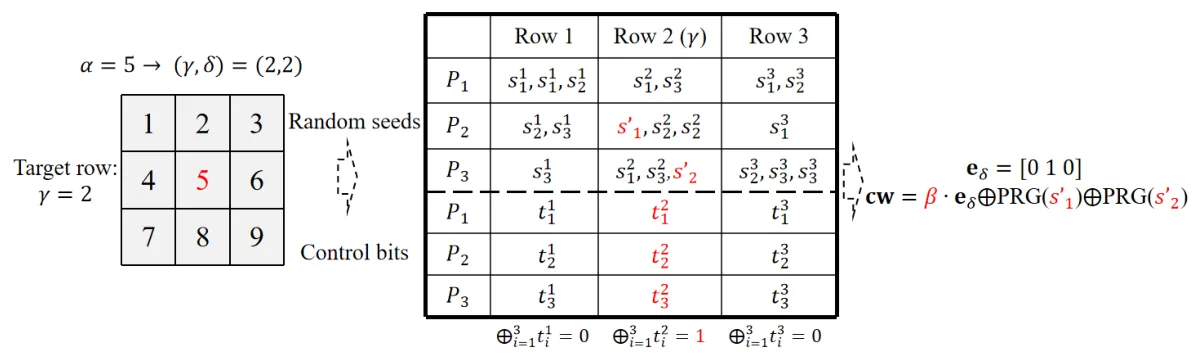
An illustrative example of the randomized construction, where N=9, α=5, p=3, ℓ=3, and r=2.

**Randomized Instantiation and Key Generation.** To distribute a point function $f_{\alpha ,\beta }$ over a domain of size *N*, the output domain is conceptually mapped to a u×u matrix, where $u=\sqrt{N}$, and the target input α corresponds to coordinate α=(γ,δ). Let $PRG:{\left\{0,1\right\}}^{\lambda }\to {\left\{0,1\right\}}^{u\cdot |F|}$ expand a seed into a vector of length *u*. For each row g∈[u], the dealer distributes PRG seeds according to the randomized bipartite graphs $G_{0}$ and $G_{1}$, where the left vertices represent the *p* computing parties and the right vertices denote the sampled seeds. A concrete example is shown in Figure 2.

As a randomized construction, for non-target rows (g≠γ), seeds are distributed via $G_{0}$, where each seed connects to exactly two randomly sampled parties (with replacement), naturally guaranteeing an even degree. For the target row (g=γ), seeds are distributed via $G_{1}$. $G_{1}$ augments the $G_{0}$ structure with *r* additional planted seeds, each connected to exactly one random party, thereby fulfilling the pseudorandomness property. Each party $P_{i}$ receives the row seeds $S_{i}^{g}$, a control bit share $t_{i}^{g}$ (where ${⨁}_{i=1}^{p}t_{i}^{g}=0$ for g≠γ, and 1 for g=γ), and a global correction word cw defined as $\mathrm{cw}=\left(\beta \cdot e_{\delta }\right){⨁}_{i\in \left[p\right],s\in S_{i}^{\gamma }}PRG\left(s\right)$. Here, $e_{\delta }$ is the unit vector with 1 at the δ-th position.

**Evaluation and Reconstruction.** During evaluation, each party $P_{i}$ independently computes the output share for row *g* via:

(1) $$y_{i}^{g}=t_{i}^{g}\cdot \mathrm{cw}\underset{s\in S_{i}^{g}}{⨁}PRG\left(s\right)$$

During reconstruction (${⨁}_{i=1}^{p}y_{i}^{g}$), by the (1−η)-Correctness property of $G_{0}$, the PRG expansions for non-target rows are evaluated an even number of times across all parties, perfectly canceling out to a zero vector. For the target row, $G_{1}$ isolates the planted degree-1 seeds, unmasking the target value $\beta \cdot e_{\delta }$ embedded within cw.

### 2.3. Replicated Secret Sharing

Let a secret *s* be an element of a finite ring $Z_{2^{l}}$. In a standard additive secret sharing (ASS) scheme [23,24,25,26], *s* is additively split into *p* shares $\left(s_{1},\ldots ,s_{p}\right)$ such that $\sum_{i=1}^{p}s_{i}=s$. This is typically achieved by sampling $s_{1},\ldots ,s_{p-1}\in Z_{2^{l}}$ uniformly at random, and computing the final share as $s_{p}=s-\sum_{i=1}^{p-1}s_{i}$. While ASS is highly efficient, it requires all *p* parties to cooperate for reconstruction, lacking fault tolerance against party dropout.

**Traditional replicated secret sharing.**

To overcome the limitation of ASS and support threshold access structures Γ where any qualified set of *t* parties can reconstruct *s*, a traditional (t,p)-threshold replicated additive secret sharing (RSS) [27] distributes multiple shares based on unqualified sets. (For notational brevity in our algorithmic descriptions, a (t,p)-threshold scheme is denoted with the subscript tofp, e.g., 2ofp). Specifically, *s* is additively split into shares $s_{T}$ such that $s=\sum_{T\in \tau }s_{T}$, where τ denotes the collection of all maximal unqualified sets of Γ (i.e., all subsets of t−1 parties). Each party i∈{1,…,p} then stores the shares $s_{T}$ for all T∈τ subject to the condition i∉T. Consequently, traditional RSS relies on exhaustive combinatorial subset allocations, requiring a substantial storage overhead of p−1t−1 shares per party.

**Figure 3:**
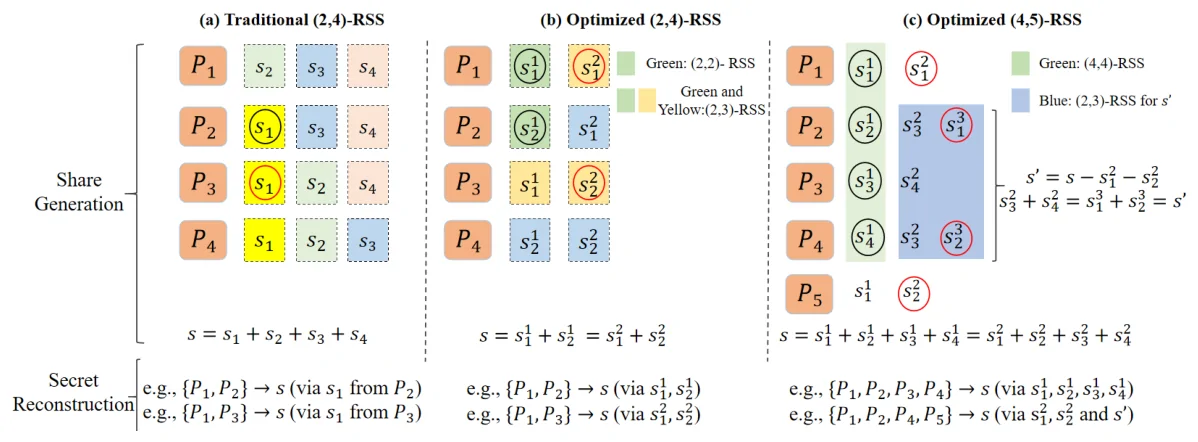
Illustrative examples of the traditional RSS and the optimized RSS.

**Example 1.** 
*Traditional (2,4)-RSS:  To illustrate, consider a traditional (2,4)-RSS as shown in Figure 3a. The maximal unqualified sets are the individual parties, yielding $\tau =\{\left\{P_{1}\right\},\left\{P_{2}\right\},\left\{P_{3}\right\},\left\{P_{4}\right\}\}$. For notational simplicity, let $s_{j}$ denote the share corresponding to the set $\left\{P_{j}\right\}$. The secret is additively split into four distinct shares ($s=s_{1}+s_{2}+s_{3}+s_{4}$). Following the storage condition i∉T, party $P_{1}$ is excluded only from $\left\{P_{1}\right\}$, meaning it must store the shares corresponding to the other three sets: $s_{2},s_{3}$, and $s_{4}$. Similarly, $P_{2}$ stores $\left\{s_{1},s_{3},s_{4}\right\}$, $P_{3}$ stores $\left\{s_{1},s_{2},s_{4}\right\}$, and $P_{4}$ stores $\left\{s_{1},s_{2},s_{3}\right\}$. Every party thus incurs a combinatorial storage overhead of 31=3 shares. For reconstruction, any two distinct parties (e.g., $P_{1}$ and $P_{2}$) simply pool their stored sets to obtain all four additive components necessary to compute $s=s_{1}+s_{2}+s_{3}+s_{4}$.*

**The optimized (2,p) incremental construction.**

In contrast to the traditional approach, we utilize an optimized RSS construction [22] that circumvents the combinatorial explosion by generating shares incrementally, ensuring identical theoretical access structures and security guarantees. The base (2,p) RSS scheme securely shares a secret *s* by generating shares as parties are added. While typically invoked as a foundational subroutine for higher-threshold variants (where t≥3), it completely defines the underlying incremental generation mechanism. For *p* parties, the dealer generates exactly $\left\lceil \log_{2}p\right\rceil$ independent share sets, where each set contains two shares summing to *s*. When a new party $P_{i}$ (i≥3) joins, its shares are determined by its corresponding conflict party $P_{conflict}$, whose index is defined as $conflict=i-2^{\left(\left\lceil \log_{2}i\right\rceil -1\right)}$. The dealer assigns the existing shares of $P_{conflict}$ to $P_{i}$. To enable $P_{conflict}$ and $P_{i}$ to jointly reconstruct the secret, the dealer samples a new share set, distributing one share to $P_{conflict}$ and the other to $P_{i}$. This construction ensures each party holds at most $\left\lceil \log_{2}p\right\rceil$ shares.

**Example 2.** 
*Optimized (2,4)-RSS: Consider constructing the optimized (2,4)-RSS as shown in Figure 3b. The dealer first generates a (2,2) ASS by sampling a first share set $\left(s_{1}^{1},s_{2}^{1}\right)$ such that $s_{1}^{1}+s_{2}^{1}=s$, providing $s_{1}^{1}$ to $P_{1}$ and $s_{2}^{1}$ to $P_{2}$. When $P_{3}$ joins, its conflict party is computed as $P_{3-2}=P_{1}$. Thus, $P_{3}$ receives $P_{1}$’s share ($s_{1}^{1}$), and a second share set $\left(s_{1}^{2},s_{2}^{2}\right)$ is generated and split between $P_{1}$ and $P_{3}$. Similarly, when $P_{4}$ joins, its conflict party is $P_{4-2}=P_{2}$. $P_{4}$ receives $P_{2}$’s share ($s_{2}^{1}$), and the dealer reuses the second share set, distributing $s_{1}^{2}$ to $P_{2}$ and $s_{2}^{2}$ to $P_{4}$. Ultimately, the parties hold $\left\{s_{1}^{1},s_{1}^{2}\right\}$, $\left\{s_{2}^{1},s_{1}^{2}\right\}$, $\left\{s_{1}^{1},s_{2}^{2}\right\}$, and $\left\{s_{2}^{1},s_{2}^{2}\right\}$ respectively. Compared to the traditional 3 shares per party, each party here only stores 2 shares. The reconstruction resolves conflicts: for instance, if the conflicting parties $P_{1}$ and $P_{3}$ cooperate, they discard their identical copied share ($s_{1}^{1}$) and sum their respective shares from the second set ($s_{1}^{2}+s_{2}^{2}=s$). Any other pair directly sums their complementary shares from the first set (e.g., $P_{1}$ and $P_{2}$ compute $s_{1}^{1}+s_{2}^{1}=s$).*

**Incremental extension to (t,p).**

Building upon the (2,p) base case, the general (t,p)-RSS scheme [22] extends the incremental share generation for an arbitrary threshold *t* through a recursive reduction process. Starting with a standard (t,t) ASS, when iteratively adding a new party $P_{i}$ (i≥t+1), the dealer identifies $P_{i-t}$ as the conflict party and copies its current shares to $P_{i}$. The dealer then samples two new shares, $s_{1}^{*}$ and $s_{2}^{*}$, sends them to $P_{i-t}$ and $P_{i}$ respectively, and computes a sub-secret $s^{\prime }=s-s_{1}^{*}-s_{2}^{*}$. The construction then recursively invokes a (t−2,i−2)-RSS to share $s^{\prime }$ among the remaining i−2 parties. This recursion reduces the threshold by 2 at each level, terminating at the boundary cases of either (2,m)- or (3,m)-RSS.

**Example 3.** 
*Optimized (4,5)-RSS: To illustrate the recursive reduction, consider a (4,5)-RSS as shown in Figure 3c. The dealer initiates a (4,4)-ASS among $P_{1},P_{2},P_{3},P_{4}$. When $P_{5}$ joins, its conflict party is $P_{5-4}=P_{1}$. The dealer copies $P_{1}$’s current shares to $P_{5}$, generates new shares $s_{1}^{2}$ and $s_{2}^{2}$ for $P_{1}$ and $P_{5}$, and computes the new sub-secret $s^{\prime }=s-s_{1}^{2}-s_{2}^{2}$. To ensure the remaining parties can collaboratively reconstruct $s^{\prime }$, the dealer recursively shares $s^{\prime }$ among $P_{2},P_{3},P_{4}$ using a (2,3)-RSS. During reconstruction, the t designated parties follow the exact reverse logic: they first resolve base-level conflicts to cooperatively reconstruct the sub-secret $s^{\prime }$, which is then added to the remaining complementary shares to recover the overarching secret s.*

## 3. Problem Formulation and Definitions

In this section, the system model and the threat model are presented. Moreover, the design goals and formal definitions of the proposed fault-tolerant DPF and PIR protocols are established.

### 3.1. System Model

**Figure 4:**
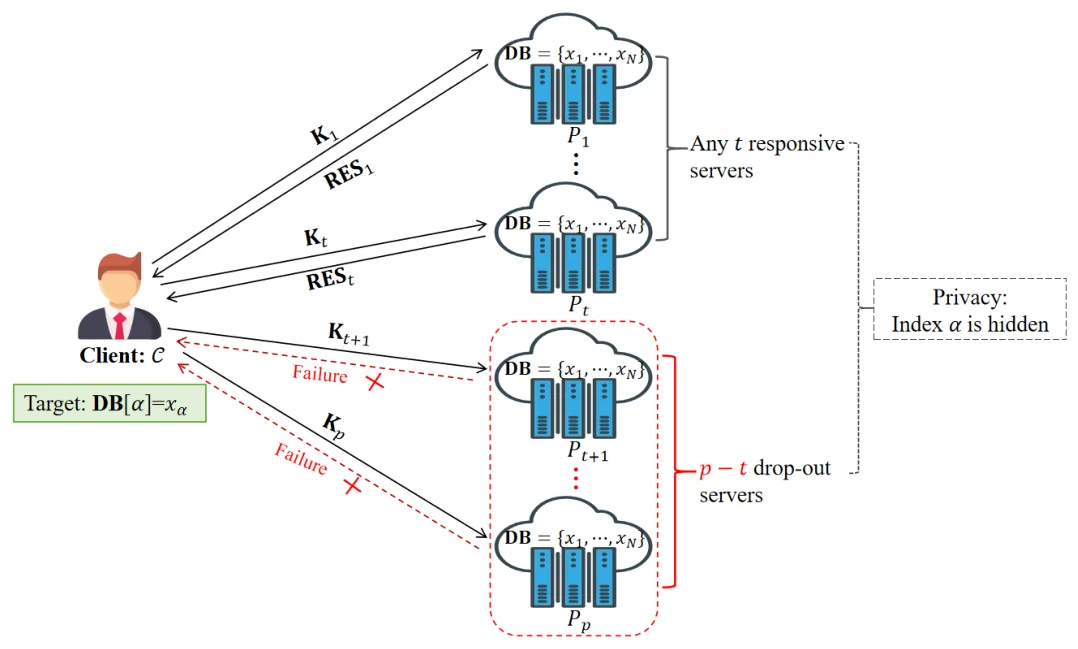
System model.

We consider a standard multi-server PIR setting comprising a single stateless client C and a set of *p* distributed servers, denoted as $P=\{P_{1},P_{2},\ldots ,P_{p}\}$. As shown in Figure 4, the servers hold identical copies of a public database DB consisting of *N* elements, i.e., $\mathrm{DB}=(x_{1},x_{2},\ldots ,x_{N})$.

When the client C wishes to retrieve the α-th element DB[α] without revealing the index α to the servers, the system execution proceeds through three primary phases:

(1) **Query Generation (PIR.Query):** The client C executes the query algorithm to create *p* shares (keys) $K_{1},\ldots ,K_{p}$. Each $K_{i}$ is transmitted to the corresponding server $P_{i}$ over a secure channel.
(2) **Evaluation (PIR.Eval):** Upon receiving the query key, each available server $P_{i}$ linearly scans the database DB and locally evaluates the DPF. It computes a partial response ${\mathrm{RES}}_{i}$ and sends it back to the client.
(3) **Crash-Resilient Reconstruction (PIR.Rec):** Due to potential network fluctuations or node failures, C may only receive a subset of responses. As long as the client collects answers from any *t* surviving servers (where t≤p), it applies the threshold reconstruction algorithm to recover the exact value of $\mathrm{DB}\left[\alpha \right]=x_{\alpha }$.

### 3.2. Threat Model

We model a distributed multi-server PIR architecture [1] comprising a single client and a cluster of *p* servers hosting replicated copies of a public database DB. To rigorously evaluate the security and robustness of the protocol, the threat model encompasses two orthogonal adversarial dimensions:

(1) **Privacy Model (Semi-Honest Collusion).** We adopt the standard honest-but-curious (semi-honest) adversarial model [28] to analyze query confidentiality. An adversary may statically corrupt and continuously monitor an unauthorized coalition of up to t−1 servers. These corrupted entities adhere to the protocol specifications (i.e., honestly executing the PIR.Eval algorithm) but attempt to infer the client’s private query index α by pooling and analyzing their designated local query shares $\left\{K_{i}\right\}$.
(2) **Availability Model (Crash Faults).** To evaluate retrieval robustness, we consider a crash fault (halting) model [29,30] during the online evaluation phase. Up to p−t servers may experience unpredictable network partitions, software exceptions, or hardware failures, rendering them completely unresponsive. We explicitly assume a non-Byzantine environment, meaning servers do not return maliciously tampered or algebraically malformed responses.
(3) **Trust Assumptions.** The client is assumed to be fully honest, properly generating query keys without colluding with any servers. Consistent with the standard PIR literature, the database DB is assumed to be public to all servers; the confidentiality of the database records themselves is outside the scope of this model.

### 3.3. Design Goals

Given the aforementioned architecture and threat model, our proposed FT-PIR scheme aims to achieve the following design goals:

(1) **Fault Tolerance (Crash-Resilient Correctness):** Even if up to p−t servers simultaneously drop out or fail to respond, the client C must still be able to output the correct element DB[α] with probability 1, relying solely on the responses from the remaining *t* available servers.
(2) **Query Privacy:** The target index α must remain computationally completely hidden from any coalition of up to t−1 colluding servers.
(3) **Communication Efficiency:** The protocol ensures a sublinear query size and completely decouples the response overhead from the database capacity *N*.
(4) **Stateless Client:** The client should require only O(1) local storage, maintaining no persistent state, cryptographic keys, or database metadata across consecutive queries.

### 3.4. Definitions of Fault-Tolerant DPF

To achieve robust resilience against server halting faults in Private Information Retrieval, we formalize the extension of the standard two-party DPF [18,19] to a generalized threshold setting. Unlike traditional DPFs that require the participation of all *p* servers to reconstruct the evaluation, a (t,p)-fault-tolerant DPF (FT-DPF) leverages a structurally redundant access policy. It guarantees correct and deterministic reconstruction from any subset of *t* surviving servers, tolerating up to p−t fail-stop faults. We formalize this primitive as follows:

**Definition 3** (Generalized (t,p)-Fault-Tolerant DPF)**.** *A (t,p)-FT-DPF scheme specialized for the Boolean point function $f_{\alpha ,1}:{\left\{0,1\right\}}^{n}\to \left\{0,1\right\}$ over the Boolean domain comprises four PPT algorithms ${\mathrm{DPF}}_{\mathrm{tofp}}=\left({\mathrm{Init}}_{\mathrm{tofp}},{\mathrm{Share}}_{\mathrm{tofp}},{\mathrm{Eval}}_{\mathrm{tofp}},{\mathrm{Rec}}_{\mathrm{tofp}}\right)$ satisfying the following properties:*

- *Syntax:*
  – *$V\leftarrow {\mathrm{Init}}_{\mathrm{tofp}}\left(t,p,1^{\lambda },f_{\alpha ,1}\right)$: A randomized setup algorithm executed by the client. It takes as input the threshold configuration (t,p), a security parameter λ, and the target Boolean point function $f_{\alpha ,1}$. It outputs the foundational base keys V.*
  – *$\left(K_{1},\ldots ,K_{p}\right)\leftarrow {\mathrm{Share}}_{\mathrm{tofp}}\left(t,p,V,P_{\mathrm{sub}}\right)$: A recursive distribution algorithm. Taking the base keys V and a reference subset $P_{\mathrm{sub}}$ (where initially $P_{\mathrm{sub}}=\left[p\right]$), it outputs a p-tuple of customized evaluation key sets $\left(K_{1},\ldots ,K_{p}\right)$, privately allocating $K_{i}$ to server i.*
  – *$Y_{i}\leftarrow {\mathrm{Eval}}_{\mathrm{tofp}}\left(i,K_{i},x\right)$: A deterministic local evaluation algorithm executed by server i. Given its index i, the local key set $K_{i}$, and an evaluation point $x\in {\left\{0,1\right\}}^{n}$, it outputs a Boolean evaluation vector $Y_{i}\in {\left\{0,1\right\}}^{F_{\max\;\mathrm{level}}\left(t,p\right)}$.*
  – *$y\leftarrow {\mathrm{Rec}}_{\mathrm{tofp}}\left(t,p,P_{\mathrm{rec}},P_{\mathrm{sub}},{\left\{Y_{i}\right\}}_{i\in P_{\mathrm{rec}}}\right)$: A deterministic iterative reconstruction algorithm. It takes as input the threshold and total server parameters (t,p), a subset of surviving server indices $P_{\mathrm{rec}}\subseteq P_{\mathrm{sub}}$ of size t, the reference subset $P_{\mathrm{sub}}$, and the corresponding evaluation vectors. It executes an iterative conflict-resolution and delegation logic to output the reconstructed Boolean scalar y∈{0,1}.*
- *Correctness: For any query target $\alpha \in {\left\{0,1\right\}}^{n}$, any evaluation point $x\in {\left\{0,1\right\}}^{n}$, and any valid subset of surviving servers $P_{\mathrm{rec}}\subseteq \left[p\right]$ with $|P_{\mathrm{rec}}|=t$, if $V\leftarrow {\mathrm{Init}}_{\mathrm{tofp}}\left(t,p,1^{\lambda },f_{\alpha ,1}\right)$ and $\left(K_{1},\ldots ,K_{p}\right)\leftarrow {\mathrm{Share}}_{\mathrm{tofp}}\left(t,p,V,\left[p\right]\right)$, then it holds that:*
(2) $$\mathrm{Pr}\left[{\mathrm{Rec}}_{\mathrm{tofp}}\left(t,p,P_{\mathrm{rec}},\left[p\right],{\left\{{\mathrm{Eval}}_{\mathrm{tofp}}\left(i,K_{i},x\right)\right\}}_{i\in P_{\mathrm{rec}}}\right)=f_{\alpha ,1}\left(x\right)\right]=1$$
- *Privacy: The scheme guarantees (t−1)-computational privacy. For any unauthorized subset of colluding server indices I⊂[p] with |I|<t, there exists a PPT simulator Sim such that the joint view of their key sets ${\left\{K_{i}\right\}}_{i\in I}$ generated by ${\mathrm{Share}}_{\mathrm{tofp}}$ is computationally indistinguishable from a simulated distribution synthesized by $\mathrm{Sim}(1^{\lambda },I)$ without knowledge of the target index α.*

### 3.5. Definitions of Fault-Tolerant PIR

Building upon the generalized FT-DPF, we construct a fault-tolerant PIR (FT-PIR) protocol that enables a client to privately retrieve a target record from a public database DB replicated across a cluster of *p* servers. The architecture guarantees both query confidentiality against semi-honest coalitions and high retrieval availability, tolerating up to p−t server halting faults during the online evaluation phase.

**Definition 4** (Generalized (t,p)-Fault-Tolerant PIR)**.**
*A (t,p)-FT-PIR scheme operating over a database of integer records DB=(DB[1],…,DB[N]) comprises three PPT algorithms PIR=(PIR.Query,PIR.Eval,PIR.Rec) satisfying the following properties:*

- *Syntax:*
  – *$\left(K_{1},\ldots ,K_{p}\right)\leftarrow \mathrm{PIR}.\mathrm{Query}\left(t,p,1^{\lambda },N,\alpha \right)$: A randomized algorithm executed by the client. It takes as input the threshold configuration (t,p), a security parameter λ, the database capacity N, and the target retrieval index α∈[N]. It initializes the reference subset $P_{\mathrm{sub}}\leftarrow \left[p\right]$, generates the base keys, and outputs a p-tuple of customized query key sets $\left(K_{1},\ldots ,K_{p}\right)$.*
  – *${\mathrm{RES}}_{j}\leftarrow \mathrm{PIR}.\mathrm{Eval}\left(j,K_{j},\mathrm{DB}\right)$: A deterministic algorithm executed locally by server j. It takes as input the server’s index j, its designated query key set $K_{j}$, and the entire database replica DB. It computes the bitwise XOR accumulation of the scalar-vector multiplications between the integer record DB[i] and its local DPF evaluation vector, outputting an aggregated response vector ${\mathrm{RES}}_{j}$ of dimension $F_{\mathrm{maxlevel}}\left(t,p\right)$.*
  – *$y\leftarrow \mathrm{PIR}.\mathrm{Rec}(t,p,P_{\mathrm{rec}},P_{\mathrm{sub}},{\left\{{\mathrm{RES}}_{i}\right\}}_{i\in P_{\mathrm{rec}}})$: A deterministic algorithm executed by the client. It takes as input the threshold and total server parameters (t,p), a subset of surviving server indices $P_{\mathrm{rec}}$ (where $|P_{\mathrm{rec}}|=t$), the reference subset $P_{\mathrm{sub}}$, and the corresponding response vectors. It executes the iterative reconstruction logic to successfully recover and output the target database record y=DB[α].*
- *Correctness: For any valid database DB of size N, any target index α∈[N], and any responsive subset of surviving servers $P_{\mathrm{rec}}\subseteq P_{\mathrm{sub}}$ with $|P_{\mathrm{rec}}|=t$, if the client computes $\left(K_{1},\ldots ,K_{p}\right)\leftarrow \mathrm{PIR}.\mathrm{Query}\left(t,p,1^{\lambda },N,\alpha \right)$, then the reconstruction algorithm deterministically recovers the target record. Formally, it holds with probability 1 that:*
(3) $$\mathrm{PIR}.\mathrm{Rec}\left(t,p,P_{\mathrm{rec}},\left[p\right],{\left\{\mathrm{PIR}.\mathrm{Eval}\left(i,K_{i},\mathrm{DB}\right)\right\}}_{i\in P_{\mathrm{rec}}}\right)=\mathrm{DB}\left[\alpha \right]$$
- *Privacy: The protocol guarantees (t−1)-computational query privacy against semi-honest adversaries. For any unauthorized coalition of server indices I⊂[p] bounded by |I|<t, there exists a PPT simulator Sim such that the joint distribution of the query key sets ${\left\{K_{i}\right\}}_{i\in I}$ generated by PIR.Query is computationally indistinguishable from a simulated distribution synthesized by $\mathrm{Sim}(1^{\lambda },N,I)$ entirely without knowledge of the client’s target index α.*

## 4. Hierarchical Construction of Threshold DPFs

In this section, we propose a (t,p)-threshold DPF tailored for PIR over the Boolean point function $f_{\alpha ,1}$. Our construction adapts the principles from RSS [22] into the FSS framework, relying solely on lightweight symmetric operations.

Our hierarchical architecture is developed in three progressive stages. First, Section 4.1 establishes the foundational (p,p)-DPF baseline. Next, Section 4.2 extends this to a (2,p) framework via an incremental extension mechanism, restricting auxiliary key expansion to a logarithmic bound ($\left\lceil \log_{2}p\right\rceil$). Finally, Section 4.3 generalizes the architecture to an arbitrary (t,p) setting. By employing recursive dimensional degradation, this generalized scheme successfully circumvents the exponential share explosion inherent in standard FSS architectures [18].

### 4.1. Base Construction: (p,p)-Threshold DPF

In this section, we present an efficient (p,p)-threshold DPF construction tailored for the Boolean point function $f_{\alpha ,1}:{\left\{0,1\right\}}^{n}\to \left\{0,1\right\}$. Building upon the foundational randomized combinatorial design proposed by [20], we introduce two critical domain-specific optimizations to significantly compress the key size: a Boolean domain restriction and an *implicit parity control* mechanism. By completely eliminating the explicit control bits required in standard constructions, our approach simultaneously reduces communication bandwidth and local key size while preserving computational privacy. The formal description of the algorithm is detailed in Algorithm 1 and elaborated below.

**Key Generation and Distribution.** To optimize the evaluation cost, the *N*-dimensional logical database is conceptually mapped into a u×u matrix, where $u=\sqrt{N}$. The target index α is projected into 2D coordinates (γ,δ)∈[u]×[u].

Unlike the general framework in [20] where the PRG outputs to a larger field space ${\left\{0,1\right\}}^{u\cdot |F|}$, we restrict our protocol to the binary field (|F|=1). Consequently, our pseudorandom generator directly expands a λ-bit seed into a *u*-bit vector (i.e., $\mathrm{PRG}:{\left\{0,1\right\}}^{\lambda }\to {\left\{0,1\right\}}^{u}$), entirely avoiding the redundant bit expansions inherent in large-field architectures.

Furthermore, to eliminate the u×p explicit correction bits required by [20], we customize the randomized bipartite graph distribution (Share). For the target row γ, the seeds are distributed via graph $G_{1}$. We mandate that the number of additional planted seeds *r*—each distributed to exactly one random server—is strictly an *odd* integer. This topological constraint inherently guarantees that the total number of surviving seeds across all *p* servers for the target row maintains an odd parity. Conversely, for non-target rows ($\gamma^{\prime }\neq \gamma$), seeds are distributed via the baseline graph $G_{0}$, where the total number of required seeds is bounded by $\ell =p^{4}\cdot \mathrm{poly}\left(\lambda \right)$, while strictly retaining an even-degree overlap constraint. Finally, the client masks the target column vector $e_{\delta }$ by constructing a single *u*-dimensional correction word cw.
**Algorithm 1** Base (p,p)-Threshold DPF for Boolean Point Function**Require:**$\mathrm{PRG}:{\left\{0,1\right\}}^{\lambda }\to {\left\{0,1\right\}}^{u}$: A pseudorandom generator, where $u=\sqrt{N}$.$e_{\delta }$: The *u*-dimensional unit vector with a 1 at the δ-th position and 0 elsewhere.Share(γ,p,u): The randomized combinatorial seed distribution algorithm (Algorithm 3 from [20]).    $\mathrm{DPF}.{\mathrm{Gen}}_{\mathrm{base}}^{p}\left(1^{\lambda },p,f_{\alpha ,1}\right)$:  1:Regard the target index α∈[N] as a coordinate pair (γ,δ)∈[u]×[u].  2:Generate PRG seed sets for all *p* servers: ${\left(S_{i}^{1},\ldots ,S_{i}^{u}\right)}_{i\in [p]}\leftarrow \mathrm{Share}\left(\gamma ,p,u\right)$.  3:Initialize the correction word $\mathrm{cw}\leftarrow e_{\delta }$.  4:**for** i=1 to *p* **do**  5:    Parse $S_{i}^{\gamma }$ as a sequence of $c_{i}$ PRG seeds $\left(s_{i}^{\gamma ,1},\ldots ,s_{i}^{\gamma ,c_{i}}\right)$.  6:    Update the correction word: $\mathrm{cw}\leftarrow \mathrm{cw}⊕\left({⨁}_{b=1}^{c_{i}}\mathrm{PRG}\left(s_{i}^{\gamma ,b}\right)\right)$.  7:**end for**  8:For each i∈[p], construct the local key: $k_{i}\leftarrow \left(\mathrm{cw}\right\|S_{i}^{1}\left\|\cdots \right\|S_{i}^{u})$.  9:**return** $\left(k_{1},\ldots ,k_{p}\right)$.$\mathrm{DPF}.{\mathrm{Eval}}_{\mathrm{base}}^{p}\left(i,k_{i},x\right)$:  1:Regard the evaluation point x∈[N] as a coordinate pair $\left(\gamma^{\prime },\delta^{\prime }\right)\in \left[u\right]\times \left[u\right]$.  2:Parse $k_{i}$ as $\left(\mathrm{cw}\|S_{i}^{1}\|\cdots \|S_{i}^{u}\right)$ and $S_{i}^{\gamma^{\prime }}$ as a sequence of $c_{i}$ seeds $\left(s_{1},\ldots ,s_{c_{i}}\right)$.  3:Compute the evaluated row vector: $d_{i}\leftarrow \left(c_{i}\;\mathrm{mod}\;2\right)\cdot \mathrm{cw}⊕\left({⨁}_{b=1}^{c_{i}}\mathrm{PRG}\left(s_{b}\right)\right)$.  4:**return** $y_{i}=d_{i}\left[\delta^{\prime }\right]$.$\mathrm{DPF}.{\mathrm{Rec}}_{\mathrm{base}}^{p}\left(y_{1},\ldots ,y_{p}\right)$:  1:**return** $y={⨁}_{i=1}^{p}y_{i}$.                                                                         ▹ Deterministically recovers $f_{\alpha ,1}\left(x\right)$

**Evaluation and Implicit Parity Logic.** During the online phase, upon receiving a query point mapped to $\left(\gamma^{\prime },\delta^{\prime }\right)$, server $P_{i}$ independently evaluates the corresponding row vector. Due to our *implicit parity control*, servers no longer rely on explicitly transmitted auxiliary bits to activate cw. Instead, the activation logic is intrinsically governed by the structural parity of the local seed count $\left(c_{i}\;\mathrm{mod}\;2\right)$. Each server directly computes its share vector as $d_{i}\leftarrow \left(c_{i}\;\mathrm{mod}\;2\right)\cdot \mathrm{cw}⊕\left({⨁}_{b=1}^{c_{i}}\mathrm{PRG}\left(s_{b}\right)\right)$.

The deterministic reconstruction relies on this topological parity:

- **Row Index Match $\left(\gamma^{\prime }=\gamma \right)$:** Graph $G_{1}$ guarantees an odd number of total surviving seeds across all active servers ($\sum c_{i}\equiv 1\;\left(\mathrm{mod}\;2\right)$). The PRG outputs perfectly cancel each other out during the XOR accumulation, cleanly unmasking the correction word cw and yielding the unit vector $e_{\delta }$.
- **Row Index Mismatch $\left(\gamma^{\prime }\neq \gamma \right)$:** Graph $G_{0}$ ensures an even seed overlap ($\sum c_{i}\equiv 0\;\left(\mathrm{mod}\;2\right)$). Consequently, both the pseudorandom expansions and the conditionally scaled cw completely cancel out, consistently producing a zero vector $0^{u}$.

This structural dependency allows the client to natively bypass the heavy algebraic interpolations typical in Shamir-based robust PIR scheme [21], replacing them with lightweight bitwise XOR aggregations.

### 4.2. Pairwise Construction: (2,p)-Threshold DPF

**Figure 5:**
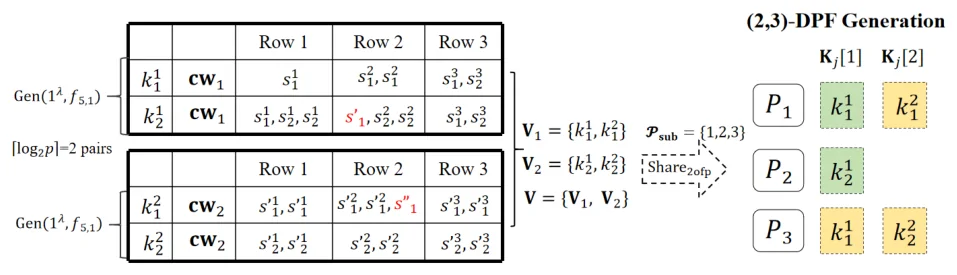
An illustrative example of the $\mathrm{DPF}.{\mathrm{Share}}_{2\mathrm{ofp}}$ key generation process (N=9, α=5, p=3, ℓ=2, r=1) detailed in Algorithm 2.

**Figure 6:**
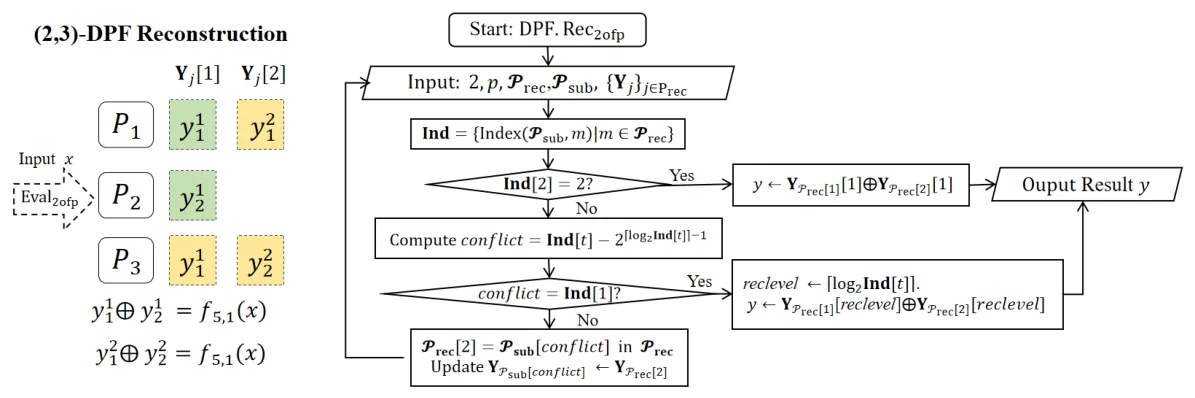
An illustrative example of the (2,3)-DPF reconstruction (N=9, α=5, p=3, ℓ=2, r=1) alongside the general workflow of $\mathrm{DPF}.{\mathrm{Rec}}_{2\mathrm{ofp}}$ detailed in Algorithm 2.

In this section, we construct an efficient (2,p)-threshold DPF scheme to address the single-point failure vulnerability inherent in strict full-participation PIR systems. This pairwise construction establishes a resilient architecture where the evaluation can be reconstructed as long as any two servers survive. Building upon the base (p,p)-DPF presented in Section 4.1 and the 2-of-*p* incremental extension mechanism proposed in [22], our scheme bypasses the exponential share blowup of traditional combinatorial designs. By precisely assigning patch keys to resolve node conflicts during incremental extension, the total number of shares per server reaches the theoretical minimum of O(logp). To intuitively demonstrate this architecture, a concrete (2,3)-DPF instantiation detailing the key generation and reconstruction phases is depicted in Figure 5 and Figure 6, respectively. The description is detailed in Algorithm 2, with the core mechanisms delineated below:
**Algorithm 2** Pairwise Construction: (2,p)-Threshold DPF via Incremental Extension**Require:**$P_{\mathrm{sub}}\subseteq \left[p\right]$: The subset of participating server indices for the current sharing phase.$P_{\mathrm{rec}}\subseteq P_{\mathrm{sub}}$: The subset of surviving servers providing responses for reconstruction, where $|P_{\mathrm{rec}}|=2$.$\mathrm{DPF}.\{{\mathrm{Gen}}_{\mathrm{base}}^{p},{\mathrm{Eval}}_{\mathrm{base}}^{p}\}$: Base algorithms imported from Algorithm 1.$\mathrm{DPF}.{\mathrm{Init}}_{2\mathrm{ofp}}\left(p,1^{\lambda },f_{\alpha ,1}\right)$:  1:**for** i=1 to $\left\lceil \log_{2}p\right\rceil$ **do**  2:    Compute base key pair: $\left(k_{1}^{\left(i\right)},k_{2}^{\left(i\right)}\right)\leftarrow \mathrm{DPF}.{\mathrm{Gen}}_{\mathrm{base}}^{p}\left(1^{\lambda },2,f_{\alpha ,1}\right)$.  3:**end for**  4:Set arrays $V_{1}\leftarrow \left(k_{1}^{\left(1\right)},\ldots ,k_{1}^{\left(\left\lceil \log_{2}p\right\rceil \right)}\right)$ and $V_{2}\leftarrow \left(k_{2}^{\left(1\right)},\ldots ,k_{2}^{\left(\left\lceil \log_{2}p\right\rceil \right)}\right)$.  5:**return** $V=(V_{1},V_{2})$.                                         ▹ Return orthogonal base patch components$\mathrm{DPF}.{\mathrm{Share}}_{2\mathrm{ofp}}\left(p,V,P_{\mathrm{sub}}\right)$:  1:Initialize local key sets $K_{P_{\mathrm{sub}}\left[i\right]}\leftarrow \emptyset$ for all i∈[p].  2:Assign baseline pair: $K_{P_{\mathrm{sub}}\left[1\right]}\leftarrow \left\{V_{1}\left[1\right]\right\}$, and $K_{P_{\mathrm{sub}}\left[2\right]}\leftarrow \left\{V_{2}\left[1\right]\right\}$.  3:**for** i=3 to *p* **do**  4:    Compute the conflict party index: $\mathrm{conflict}=i-2^{\lceil \log_{2}i\rceil -1}$.  5:    Inherit state: $K_{P_{\mathrm{sub}}\left[i\right]}\leftarrow K_{P_{\mathrm{sub}}\left[\mathrm{conflict}\right]}$.                         ▹ Inherit state from the conflict party  6:    Resolve conflict: Add $V_{1}\left[\left\lceil \log_{2}i\right\rceil \right]$ to $K_{P_{\mathrm{sub}}\left[\mathrm{conflict}\right]}$, and $V_{2}\left[\left\lceil \log_{2}i\right\rceil \right]$ to $K_{P_{\mathrm{sub}}\left[i\right]}$.  7:**end for**  8:**return** $\left(K_{P_{\mathrm{sub}}\left[1\right]},\ldots ,K_{P_{\mathrm{sub}}\left[p\right]}\right)$.$\mathrm{DPF}.{\mathrm{Eval}}_{2\mathrm{ofp}}\left(j,K_{j},x\right)$:  1:Parse local key set $K_{j}$ as a sequence of base keys $\left(k_{j,1},k_{j,2},\ldots ,k_{j,|K_{j}|}\right)$.  2:**for** each $i\in [|K_{j}|]$ **do**  3:    Compute local bit share: $y_{j,i}\leftarrow \mathrm{DPF}.{\mathrm{Eval}}_{\mathrm{base}}^{p}\left(j,k_{j,i},x\right)$.  4:**end for**  5:**return** the aggregated share vector $Y_{j}=\left(y_{j,1},y_{j,2},\ldots ,y_{j,|K_{j}|}\right)$.$\mathrm{DPF}.{\mathrm{Rec}}_{2\mathrm{ofp}}\left(2,p,P_{\mathrm{rec}},P_{\mathrm{sub}},{\left\{Y_{j}\right\}}_{j\in P_{\mathrm{rec}}}\right)$:  1:Initialize output bit y←0.  2:Map surviving servers to their relative 1-based indices within $P_{\mathrm{sub}}$: $\mathrm{Ind}\leftarrow (\mathrm{Index}\left(P_{\mathrm{sub}},P_{\mathrm{rec}}\left[1\right]\right),\mathrm{Index}\left(P_{\mathrm{sub}},P_{\mathrm{rec}}\left[2\right]\right))$.  3:**while true do**  4:    **if** Ind[2]=2 **then**  5:        $y\leftarrow Y_{P_{\mathrm{rec}}\left[1\right]}\left[1\right]⊕Y_{P_{\mathrm{rec}}\left[2\right]}\left[1\right]$; **break**.                                          ▹ Baseline pair resolution  6:    **else**  7:        $\mathrm{conflict}=\mathrm{Ind}\left[2\right]-2^{\lceil \log_{2}\left(\mathrm{Ind}\left[2\right]\right)\rceil -1}$.  8:        **if** conflict=Ind[1] **then**  9:           $\mathrm{reclevel}\leftarrow \lceil \log_{2}\left(\mathrm{Ind}\left[2\right]\right)\rceil$.10:           $y\leftarrow Y_{P_{\mathrm{rec}}\left[1\right]}\left[\mathrm{reclevel}\right]⊕Y_{P_{\mathrm{rec}}\left[2\right]}\left[\mathrm{reclevel}\right]$; **break**.         ▹ Target conflict level resolution11:        **else**12:           Replace $P_{\mathrm{rec}}\left[2\right]$ with $P_{\mathrm{sub}}\left[\mathrm{conflict}\right]$ in $P_{\mathrm{rec}}$, and re-sort ascendingly.13:           $Y_{P_{\mathrm{sub}}\left[\mathrm{conflict}\right]}\leftarrow Y_{P_{\mathrm{rec}}\left[2\right]}$.                                       ▹ Conflict party response substitution14:           Update Ind based on the newly sorted $P_{\mathrm{rec}}$.15:        **end if**16:    **end if**17:**end while**18:**return** *y*.                                                                             ▹ Deterministically recovers $f_{\alpha ,1}\left(x\right)$

**Initialization and Component Generation.** Instead of computing a monolithic sharing scheme, the algorithm pre-generates foundational components by invoking the base algorithm $\mathrm{DPF}.{\mathrm{Gen}}_{\mathrm{base}}^{p}$ exactly $\left\lceil \log_{2}p\right\rceil$ times. This initialization produces fully independent base (2,2)-DPF key pairs, denoted as sequences $V_{1}$ and $V_{2}$. These independent base keys serve as the fundamental patch components for the subsequent incremental allocation, ensuring that cryptographic conflicts at distinct hierarchical levels are resolved entirely independently.

**Incremental Allocation.** The pre-generated base keys are distributed among the *p* servers using a dynamic inheritance mechanism within a target subset $P_{\mathrm{sub}}$. The distribution begins by establishing a baseline (2,2) association, assigning $V_{1}\left[1\right]$ to $P_{\mathrm{sub}}\left[1\right]$ and $V_{2}\left[1\right]$ to $P_{\mathrm{sub}}\left[2\right]$. For any newly appended server $P_{\mathrm{sub}}\left[i\right]$ (i≥3), the scheme determines its conflict party index via $\mathrm{conflict}=i-2^{\lceil \log_{2}i\rceil -1}$.

To establish the required structural redundancy, $P_{\mathrm{sub}}\left[i\right]$ first duplicates the entire existing key set of its conflict party $P_{\mathrm{sub}}\left[\mathrm{conflict}\right]$. Crucially, to resolve the cryptographic conflict (since $P_{i}$ and $P_{\mathrm{conflict}}$ now hold identical shares and cannot reconstruct a secret together), the client injects a fresh set of patch keys at the current recursive level $\left\lceil \log_{2}i\right\rceil$: $V_{1}\left[\left\lceil \log_{2}i\right\rceil \right]$ is assigned to $P_{\mathrm{sub}}\left[\mathrm{conflict}\right]$, while $V_{2}\left[\left\lceil \log_{2}i\right\rceil \right]$ is assigned to $P_{\mathrm{sub}}\left[i\right]$. This precisely tailored patching establishes a new pairwise association without disrupting previous layers, culminating in *p* logarithmic-size key sets.

**Remark 1.** 
*Connection to Evolving Secret Sharing (ESS). Unlike pure ESS [31], which dynamically scales without predefined limits or updates to existing shares, our approach is a threshold-constrained incremental construction. By relying on a bounded cluster size p and utilizing patch keys to update specific conflict parties, we intentionally trade the non-update property of pure ESS to achieve the theoretical minimum of O(logp) share size.*

**Evaluation and Iterative Conflict Resolution.** During the online phase, upon receiving a query *x*, each server $P_{j}$ evaluates all base keys within its local set $K_{j}$ in parallel, yielding a compact evaluated share vector $Y_{j}$.

The fault-tolerant reconstruction logic is executed by the client upon receiving share vectors $Y_{P_{\mathrm{rec}}\left[1\right]}$ and $Y_{P_{\mathrm{rec}}\left[2\right]}$ from any two surviving servers. After mapping the active servers to their 1-based indices within the current participating subset $P_{\mathrm{sub}}$ (assuming Ind[1]<Ind[2]), the client executes an *iterative conflict resolution* mechanism:

- **Baseline Resolution:** If Ind[2]=2, the responding servers correspond exactly to the foundational baseline pair established at the root of the incremental topology. The client deterministically recovers the point function value by directly extracting and XOR-summing the first elements of their respective share vectors (i.e., $Y_{P_{\mathrm{rec}}\left[1\right]}\left[1\right]⊕Y_{P_{\mathrm{rec}}\left[2\right]}\left[1\right]$).
- **Direct Conflict Resolution:** If Ind[1] is the direct conflict party of Ind[2] (i.e., $\mathrm{Ind}\left[1\right]=\mathrm{conflict}=\mathrm{Ind}\left[2\right]-2^{\lceil \log_{2}\left(\mathrm{Ind}\left[2\right]\right)\rceil -1}$), the structural conflict was resolved precisely at level $\left\lceil \log_{2}\left(\mathrm{Ind}\left[2\right]\right)\right\rceil$. The client extracts the corresponding bits from $Y_{P_{\mathrm{rec}}\left[1\right]}$ and $Y_{P_{\mathrm{rec}}\left[2\right]}$ at this specific level and computes the XOR sum to recover $f_{\alpha ,1}\left(x\right)$.
- **Conflict Tracing:** If the responding servers are neither the baseline pair nor a direct conflict pair, the client iteratively replaces the larger index $P_{\mathrm{rec}}\left[2\right]$ with its corresponding conflict party $P_{\mathrm{sub}}\left[\mathrm{conflict}\right]$, re-sorts the active set, and substitutes the response vector. This deterministic traversal iteratively steps back through the allocation topology until a direct conflict pair or the baseline pair is reached.

### 4.3. Generalized Construction: (t,p)-Threshold DPF

To accommodate diverse fault-tolerance requirements, we now generalize the pairwise construction to an arbitrary (t,p)-threshold scenario by integrating the base (t,t)-DPF with the *t*-of-*p* recursive patching mechanism [22]. It systematically reduces high-order threshold conflicts into lower-order (t−2) sub-problems through recursive dimensional degradation, guaranteeing (t−1)-privacy while maintaining polynomial scalability. Specifically, the recursion depth and the maximum share expansion per server are bounded by the piecewise function $F_{\mathrm{maxlevel}}\left(t,p\right)$, defined as:

(4) $$F_{\mathrm{maxlevel}}\left(t,p\right)=\left\{\begin{array}{cc} \lceil \log_{2}p\rceil , & \mathrm{if}\;t=2\\ p-2, & \mathrm{if}\;t=3\\ 1, & \mathrm{if}\;t=p\\ F_{\mathrm{maxlevel}}\left(t,p-1\right)+F_{\mathrm{maxlevel}}\left(t-2,p-2\right), & \mathrm{otherwise} \end{array}\right.$$

Note that the asymptotic growth of $F_{\mathrm{maxlevel}}\left(t,p\right)$ depends on the relationship between the threshold *t* and the number of servers *p*. For a fixed constant *t*, it is polynomially bounded by $O(p^{(t-2)/2}\log p)$ for even t≥2, and $O(p^{(t-1)/2})$ for odd t≥3. Conversely, if *t* scales linearly with *p* (e.g., t=Θ(p)), the recursion induces an exponential combinatorial expansion. We therefore utilize $F_{\mathrm{maxlevel}}\left(t,p\right)$ as a precise parameterized bound to explicitly capture this dynamic, rather than assuming a generic polynomial jointly in *t* and *p*. Since practical PIR deployments typically restrict *t* to a small constant (N≫p≥t), the protocol strictly operates within this efficient polynomial regime.

**Figure 7:**
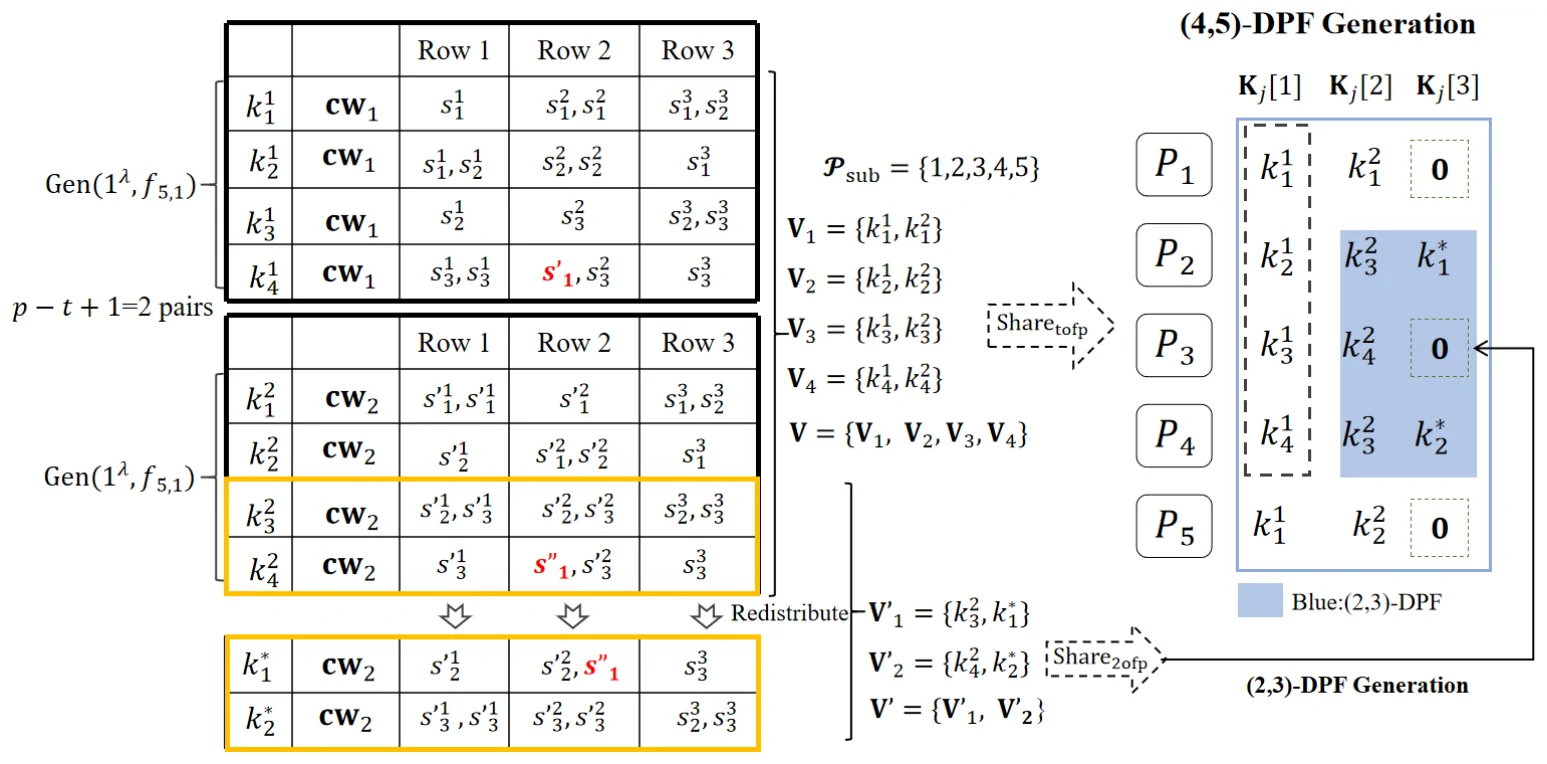
An illustrative example of the $\mathrm{DPF}.{\mathrm{Share}}_{\mathrm{tofp}}$ key generation process (N=9, α=5, p=5, t=4, ℓ=3, r=1) detailed in Algorithm 3.

**Figure 8:**
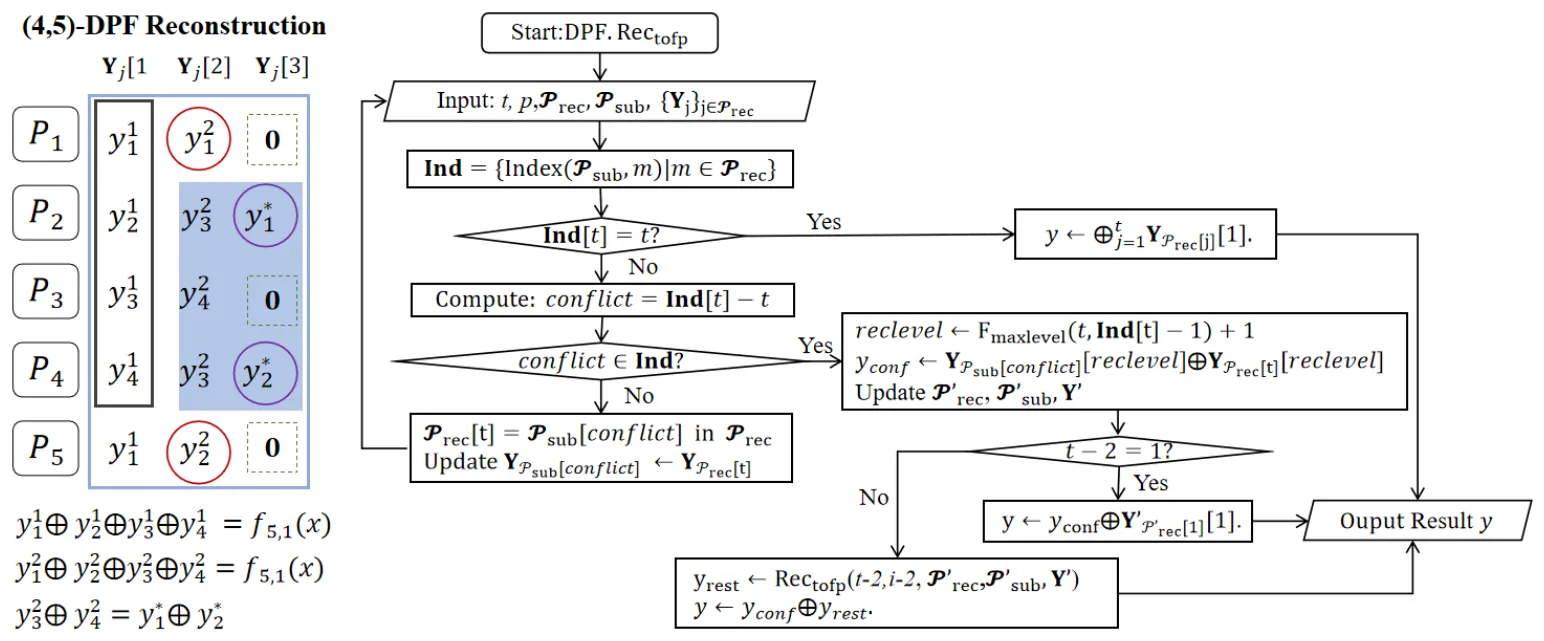
An illustrative example of the (4,5)-DPF reconstruction (N=9, α=5, p=5, t=4, ℓ=3, r=1) alongside the general workflow of $\mathrm{DPF}.{\mathrm{Rec}}_{\mathrm{tofp}}$ detailed in Algorithm 4.

A visual intuition of this construction, illustrating the incremental key generation and fault-tolerant reconstruction for a concrete (4,5) setting, is provided in Figure 7 and Figure 8, respectively. The procedures are divided and detailed in Algorithms 3 and 4, with the core design paradigm delineated below:

**Initialization and Component Grouping (Algorithm 3).** Rather than relying on a single monolithic generation, the algorithm pre-computes p−t+1 independent base (t,t)-DPF key sets. These foundational blocks are grouped into *t* independent sequences $V_{1},\ldots ,V_{t}$. Compared to the pairwise scheme, this high-dimensional initialization supports *t*-party reconstruction.
**Algorithm 3** Generalized (t,p)-Threshold DPF: Setup and Sharing**Require:**$\mathrm{DPF}.{\mathrm{Gen}}_{\mathrm{base}}^{t}$: Base generation algorithm instantiated for *t* parties from Algorithm 1.$\mathrm{DPF}.{\mathrm{Share}}_{2\mathrm{ofp}}$: Pairwise sharing imported from Algorithm 2.$F_{\mathrm{maxlevel}}\left(t,p\right)$: Maximum sharing level function defined in Equation (4).    $\mathrm{DPF}.{\mathrm{Init}}_{\mathrm{tofp}}\left(t,p,1^{\lambda },f_{\alpha ,1}\right)$:  1:**for** each i∈[p−t+1] **do**  2:    Compute base key set: $\left(k_{1}^{i},k_{2}^{i},\ldots ,k_{t}^{i}\right)\leftarrow \mathrm{DPF}.{\mathrm{Gen}}_{\mathrm{base}}^{t}\left(1^{\lambda },t,f_{\alpha ,1}\right)$.    ▹ Generate p−t+1 independent sets of *t*-party base keys.  3:**end for**  4:Set arrays $V_{j}\leftarrow \left(k_{j}^{1},k_{j}^{2},\ldots ,k_{j}^{p-t+1}\right)$ for all j∈[t]. ▹ Group the independent base components for party *j*.  5:**return** $V=(V_{1},\ldots ,V_{t})$.$\mathrm{DPF}.{\mathrm{Share}}_{\mathrm{tofp}}\left(t,p,V,P_{\mathrm{sub}}\right)$:  1:Initialize local key sets $K_{P_{\mathrm{sub}}\left[i\right]}\leftarrow \emptyset$ for all i∈[p].  2:**if** t=2 **then**  3:    **return** $\left(K_{1},\ldots ,K_{p}\right)\leftarrow \mathrm{DPF}.{\mathrm{Share}}_{2\mathrm{ofp}}\left(p,V,P_{\mathrm{sub}}\right)$.  4:**end if**  5:Assign foundational shares: Add $V_{j}\left[1\right]$ to $K_{P_{\mathrm{sub}}\left[j\right]}$ for all j∈[t].  6:**for** i=t+1 to *p* **do**  7:    conflict=i−t.                                                   ▹ Calculate the conflict index for high-order sharing.  8:    Inherit state: $K_{P_{\mathrm{sub}}\left[i\right]}\leftarrow K_{P_{\mathrm{sub}}\left[\mathrm{conflict}\right]}$.                                   ▹ Duplicate keys from the conflict party.  9:    Resolve primary conflict: Add $V_{1}\left[i-t+1\right]$ to $K_{P_{\mathrm{sub}}\left[\mathrm{conflict}\right]}$ and $V_{2}\left[i-t+1\right]$ to $K_{P_{\mathrm{sub}}\left[i\right]}$.10:    Define target subset for degradation: $P_{\mathrm{sub}}^{\prime }\leftarrow \{P_{\mathrm{sub}}\left[m\right]\in P_{\mathrm{sub}}|m<i$ and m≠conflict}.11:    **if** t−2=1 **then**                                                                                        ▹ Boundary condition: t=312:        **for** each j∈[i−2] **do**13:           Add $V_{3}\left[i-t+1\right]$ to $K_{P_{\mathrm{sub}}^{\prime }\left[j\right]}$.                             ▹ Distribute patch key to the remaining nodes.14:        **end for**15:    **else**                                                                                                         ▹ Recursive degradation: t≥416:        For each j∈[t−2], parse $V_{j+2}\left[i-t+1\right]$ as $\left(\mathrm{cw}\|S_{j}^{1}\|\cdots \|S_{j}^{u}\right)$.17:        For each g∈[u], let $S^{\prime g}=\left\{S_{3}^{g},S_{4}^{g},\ldots ,S_{t}^{g}\right\}$.                                                         ▹ Extract row seeds.18:        Let $L=\left\{\begin{array}{cc} \left\lceil \log_{2}\left(i-2\right)\right\rceil & \;\mathrm{if}\;t-2=2\\ i-t+1 & \mathrm{otherwise} \end{array}\right.$.                            ▹ Determine recursive generation depth.19:        Initialize first subkey set: ${\left\{k_{j}^{\prime }\right\}}^{\left(1\right)}\leftarrow V_{j+2}\left[i-t+1\right]$ for j∈[t−2].20:        **for** b=2 to *L* **do**                                                              ▹ Generate combinatorial permutations.21:           For each g∈[u], randomly permute and split $S^{\prime g}$ into t−2 distinct shares: $S_{1}^{\prime g},\ldots ,S_{t-2}^{\prime g}$.22:           For each j∈[t−2], construct subkey: ${\left\{k_{j}^{\prime }\right\}}^{\left(b\right)}\leftarrow \left(\mathrm{cw}\right\|S_{j}^{\prime 1}\|S_{j}^{\prime 2}\left\|\cdots \right\|S_{j}^{\prime u})$.23:        **end for**24:        For each j∈[t−2], group patched subkeys: $V_{j}^{\prime }\leftarrow \left({\left\{k_{j}^{\prime }\right\}}^{\left(1\right)},{\left\{k_{j}^{\prime }\right\}}^{\left(2\right)},\ldots ,{\left\{k_{j}^{\prime }\right\}}^{\left(L\right)}\right)$.25:        Let $V^{\prime }=\left(V_{1}^{\prime },\ldots ,V_{t-2}^{\prime }\right)$.26:        Recursively distribute shares: $\left(K_{1}^{\prime },\ldots ,K_{i-2}^{\prime }\right)\leftarrow \mathrm{DPF}.{\mathrm{Share}}_{\mathrm{tofp}}\left(t-2,i-2,V^{\prime },P_{\mathrm{sub}}^{\prime }\right)$.27:        For each $m\in P_{\mathrm{sub}}^{\prime }$, append keys: $K_{m}\leftarrow K_{m}\|K_{m}^{\prime }$.28:        $\ell_{\max}\leftarrow \max_{m\in [i]}\left|K_{P_{\mathrm{sub}}\left[m\right]}\right|$.                                    ▹ Determine the maximum number of elements.29:        For each m∈[i], pad keys: $K_{P_{\mathrm{sub}}\left[m\right]}\leftarrow K_{P_{\mathrm{sub}}\left[m\right]}\|0^{\ell_{\mathrm{maxlevel}}-\left|K_{P_{\mathrm{sub}}\left[m\right]}\right|}$. ▹ Zero-padding for memory alignment.30:    **end if**31:**end for**32:**return** $\left(K_{P_{\mathrm{sub}}\left[1\right]},\ldots ,K_{P_{\mathrm{sub}}\left[p\right]}\right)$.

**Recursive Share Allocation (Algorithm 3).** The distribution leverages a dynamic inheritance and patching mechanism to output *p* customized key sets. For the base configuration (t=2), the procedure directly falls back to the pairwise allocation logic. For higher thresholds (t>2), the algorithm first assigns foundational keys to the initial *t* servers. For any incrementally added server $P_{\mathrm{sub}}\left[i\right]$ (i>t), the allocation proceeds in two critical phases:

1. *Conflict Inheritance and Resolution:* The new server $P_{\mathrm{sub}}\left[i\right]$ first inherits the complete structural state of its corresponding conflict party $P_{\mathrm{sub}}\left[\mathrm{conflict}\right]$ (where conflict=i−t). Subsequently, fresh patch components from $V_{1}$ and $V_{2}$ are assigned to both parties respectively, precisely resolving the primary cryptographic conflict at that level.
2. *Dimensional Degradation:* For the remaining t−2 components, the scheme applies a polynomial-size combinatorial permutation to re-partition the PRG seeds. By recursively invoking the allocation function, the architecture elegantly collapses the remaining high-order sharing requirements into a manageable (t−2)-of-(i−2) sub-problem. As visualized in Figure 7, auxiliary patch keys are precisely generated at the specified hierarchical depth.

**Algorithm 4** Generalized (t,p)-Threshold DPF: Evaluation and Reconstruction**Require:**$\mathrm{DPF}.\{{\mathrm{Eval}}_{2\mathrm{ofp}},{\mathrm{Rec}}_{2\mathrm{ofp}}\}$: Pairwise algorithms imported from Algorithm 2.$P_{\mathrm{rec}}$: A sorted list of *t* surviving server indices.$F_{\mathrm{maxlevel}}\left(t,p\right)$: Maximum sharing level function defined in Equation (4).    $\mathrm{DPF}.{\mathrm{Eval}}_{\mathrm{tofp}}\left(j,K_{j},x\right)$:  1:$Y_{j}\leftarrow \mathrm{DPF}.{\mathrm{Eval}}_{2\mathrm{ofp}}\left(j,K_{j},x\right)$.         ▹ Local evaluation intrinsically matches the pairwise logic.  2:**return** $Y_{j}$.$\mathrm{DPF}.{\mathrm{Rec}}_{\mathrm{tofp}}\left(t,p,P_{\mathrm{rec}},P_{\mathrm{sub}},{\left\{Y_{i}\right\}}_{i\in P_{\mathrm{rec}}}\right)$:  1:Initialize output bit y←0.  2:**if** t=2 **then**  3:    **return** $\mathrm{DPF}.{\mathrm{Rec}}_{2\mathrm{ofp}}\left(2,p,P_{\mathrm{rec}},P_{\mathrm{sub}},{\left\{Y_{j}\right\}}_{j\in P_{\mathrm{rec}}}\right)$.  4:**end if**  5:Map surviving servers to relative 1-based indices: $\mathrm{Ind}\leftarrow (\mathrm{Index}\left(P_{\mathrm{sub}},m\right)|m\in P_{\mathrm{rec}})$.  6:**while true do**  7:    **if** Ind[t]=t **then**  8:        $y\leftarrow {⨁}_{j=1}^{t}Y_{P_{\mathrm{rec}}\left[j\right]}\left[1\right]$; **break**.                ▹ Base case: XOR first shares of baseline servers.  9:    **else**10:        conflict←Ind[t]−t.                               ▹ Calculate the conflict index for server $P_{\mathrm{rec}}\left[t\right]$.11:        **if** conflict∈Ind **then**12:           $\mathrm{reclevel}\leftarrow F_{\mathrm{maxlevel}}\left(t,\mathrm{Ind}\left[t\right]-1\right)+1$.                      ▹ Determine the active target level.13:           $y_{\mathrm{conf}}\leftarrow Y_{P_{\mathrm{sub}}\left[\mathrm{conflict}\right]}\left[\mathrm{reclevel}\right]⊕Y_{P_{\mathrm{rec}}\left[t\right]}\left[\mathrm{reclevel}\right]$.                  ▹ Primary conflict resolution.14:           $P_{\mathrm{rec}}^{\prime }\leftarrow P_{\mathrm{rec}}\setminus \left\{P_{\mathrm{sub}}\left[\mathrm{conflict}\right],P_{\mathrm{rec}}\left[t\right]\right\}$.15:           $P_{\mathrm{sub}}^{\prime }\leftarrow \left\{m\in P_{\mathrm{sub}}|m<P_{\mathrm{rec}}\left[t\right]\;\mathrm{and}\;m\neq P_{\mathrm{sub}}\left[\mathrm{conflict}\right]\right\}$.16:           **for** each $j\in P_{\mathrm{rec}}^{\prime }$ **do**17:               Extract suffix vector: $Y_{j}^{\prime }\leftarrow Y_{j}\left[\mathrm{reclevel}\ldots \right|Y_{j}\left|\right]$.                     ▹ Prune evaluated shares.18:           **end for**19:           **if** t−2=1 **then**                                                                     ▹ Boundary condition: t=320:               $y\leftarrow y_{\mathrm{conf}}⊕Y_{P_{\mathrm{rec}}^{\prime }\left[1\right]}^{\prime }\left[1\right]$; **break**. ▹ Extract the first share bit from the single remaining server.21:           **else**                                                                 ▹ Recursive degradation resolution: t≥422:               $y_{\mathrm{rest}}\leftarrow \mathrm{DPF}.{\mathrm{Rec}}_{\mathrm{tofp}}\left(t-2,\right|P_{\mathrm{sub}}^{\prime }\left|,P_{\mathrm{rec}}^{\prime },P_{\mathrm{sub}}^{\prime },{\left\{Y_{i}^{\prime }\right\}}_{i\in P_{\mathrm{rec}}^{\prime }}\right)$.23:               $y\leftarrow y_{\mathrm{conf}}⊕y_{\mathrm{rest}}$; **break**.24:           **end if**25:        **else**26:           $Y_{P_{\mathrm{sub}}\left[\mathrm{conflict}\right]}\leftarrow Y_{P_{\mathrm{rec}}\left[t\right]}$.                                         ▹ Conflict party response substitution.27:           Replace $P_{\mathrm{rec}}\left[t\right]$ with $P_{\mathrm{sub}}\left[\mathrm{conflict}\right]$ in $P_{\mathrm{rec}}$, and re-sort ascendingly.28:           Update Ind based on the newly sorted $P_{\mathrm{rec}}$.               ▹ Synchronize index mapping.29:        **end if**30:    **end if**31:**end while**32:**return** *y*.                                                                               ▹ Deterministically recovers $f_{\alpha ,1}\left(x\right)$.

**Evaluation and Iterative Reconstruction (Algorithm 4).** During the online phase, each server evaluates its local key set in parallel. The reconstruction algorithm performs collaborative fault-tolerant recovery based on the set of *t* surviving servers $P_{\mathrm{rec}}$ (as illustrated in Figure 8):

- *Base Case (t=2):* The function directly invokes the pairwise base reconstruction logic.
- *Iterative Traversal and Conflict Resolution (t>2):* The algorithm first maps each element in the surviving set to its relative 1-based index array Ind within the current participating subset $P_{\mathrm{sub}}$. It then iteratively traverses the sharing topology via a deterministic loop:
  – **Base t-party Subset:** If the maximum current index satisfies Ind[t]=t, the foundational base subset is reached. The target bit is recovered directly by XOR-accumulating the first elements of the evaluation shares from these initial *t* servers.
  – **Direct Conflict Resolution:** It computes the conflict index conflict=Ind[t]−t. If conflict∈Ind, a direct structural conflict is identified. The algorithm determines the target active level reclevel, extracts the primary resolution $y_{\mathrm{conf}}$ by XORing the corresponding shares, prunes the evaluated suffix vectors, and reduces the operation into a (t−2) sub-problem. (If t−2=1, it directly extracts the scalar bit; otherwise, it resolves recursively).
  – **Conflict Party Substitution:** If conflict∉Ind, it implies the conflict party is absent and its role must be delegated. The algorithm performs a response substitution: the highest-index active server $P_{\mathrm{rec}}\left[t\right]$ is replaced by its conflict party $P_{\mathrm{sub}}\left[\mathrm{conflict}\right]$. The active set is then re-sorted, and the index mapping Ind is synchronized to continue the iterative traversal towards the base subset.

## 5. The Proposed Fault-Tolerant PIR Scheme

**Figure 9:**
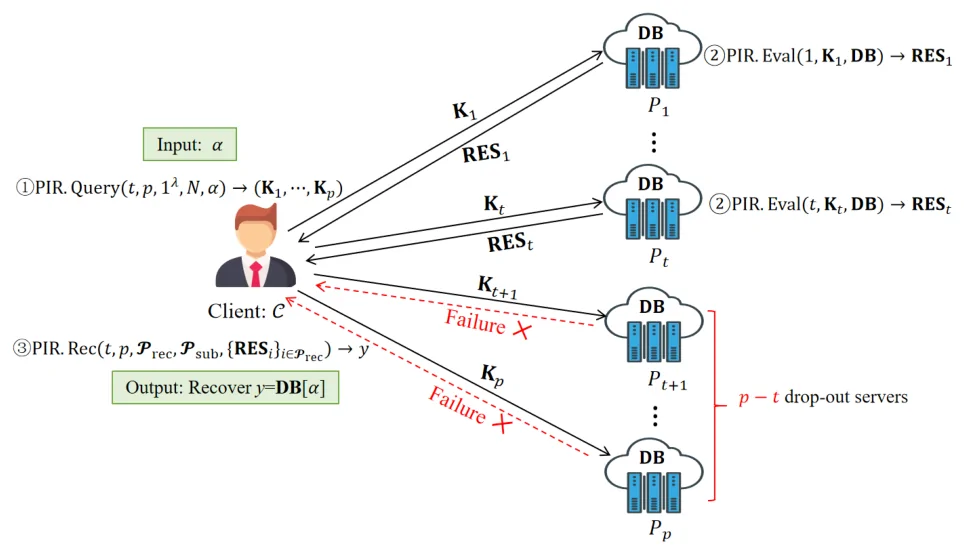
Workflow of the proposed PIR.

Building upon the generalized (t,p)-threshold DPF constructed in Section 4, we now formalize our robust PIR scheme. The protocol proceeds in three standard phases: Query, Evaluation, and Reconstruction, as detailed in Algorithm 5 and visually summarized in Figure 9. Let the database be modeled as an array of *N* records DB=(DB[1],…,DB[N]), where each record DB[i] is treated as an integer value.
**Algorithm 5** Fault-Tolerant PIR Based on Generalized (t,p)-Threshold DPF**Notation:** We utilize the generalized DPF algorithms $\mathrm{DPF}.\{{\mathrm{Init}}_{\mathrm{tofp}},{\mathrm{Share}}_{\mathrm{tofp}},{\mathrm{Eval}}_{\mathrm{tofp}},{\mathrm{Rec}}_{\mathrm{tofp}}\}$ established in Algorithms 3 and 4. $F_{\mathrm{maxlevel}}\left(t,p\right)$ is the maximum sharing level function defined in Equation (4). The database contains *N* records, formulated as DB=(DB[1],…,DB[N]).    **Input:** Client’s target index α∈[N].**Output:** The retrieved record DB[α].    $\mathrm{PIR}.\mathrm{Query}(t,p,1^{\lambda },N,\alpha )$:  1:Let $P_{\mathrm{sub}}\leftarrow \left[p\right]$.                     ▹ Initialize the reference subset to include all *p* servers.  2:Generate base keys: $V\leftarrow {\mathrm{Init}}_{\mathrm{tofp}}\left(t,p,1^{\lambda },f_{\alpha ,1}\right)$.  3:Distribute via recursive patching: $\left(K_{1},\ldots ,K_{p}\right)\leftarrow {\mathrm{Share}}_{\mathrm{tofp}}\left(t,p,V,P_{\mathrm{sub}}\right)$.  4:**return** query sets $\left(K_{1},\ldots ,K_{p}\right)$ to servers 1,…,p, respectively.$\mathrm{PIR}.\mathrm{Eval}(j,K_{j},\mathrm{DB})$:  1:Initialize ${\mathrm{RES}}_{j}\leftarrow 0$.                     ▹ Allocate a zero vector of dimension $F_{\mathrm{maxlevel}}\left(t,p\right)$.  2:**for** i=1 to *N* **do**  3:    Extract share vector for the current index: $Y_{j}^{\left(i\right)}\leftarrow {\mathrm{Eval}}_{\mathrm{tofp}}\left(j,K_{j},i\right)$.  4:    Accumulate response: ${\mathrm{RES}}_{j}\leftarrow {\mathrm{RES}}_{j}⊕\left(Y_{j}^{\left(i\right)}\cdot \mathrm{DB}\left[i\right]\right)$. ▹ Scalar-vector multiplication with integer DB[i].  5:**end for**  6:**return** ${\mathrm{RES}}_{j}$.$\mathrm{PIR}.\mathrm{Rec}(t,p,P_{\mathrm{rec}},P_{\mathrm{sub}},{\left\{{\mathrm{RES}}_{i}\right\}}_{i\in P_{\mathrm{rec}}})$:  1:Execute iterative reconstruction: $y\leftarrow {\mathrm{Rec}}_{\mathrm{tofp}}\left(t,p,P_{\mathrm{rec}},P_{\mathrm{sub}},{\left\{{\mathrm{RES}}_{i}\right\}}_{i\in P_{\mathrm{rec}}}\right)$.  2:**return** *y*.                                                                           ▹ Successfully recover y=DB[α].

### 5.1. Protocol Description

(1) **Query Generation (**PIR.Query**).** To privately retrieve the target record without revealing its index α to the servers, the client executes a PIR request. Specifically, the client initializes the reference subset $P_{\mathrm{sub}}\leftarrow \left[p\right]$ and encapsulates α into a set of cryptographic query keys via the (t,p)-DPF base generation algorithm. It then distributes the query shares $\left(K_{1},\ldots ,K_{p}\right)$ to the respective servers. The communication cost in this phase is precisely bounded by the key expansion of the (t,p)-DPF.
(2) **Server Evaluation (**PIR.Eval**).** Upon receiving the query share $K_{j}$, server *j* expands it into a local secret-shared indicator vector. The server then evaluates the query by computing the inner product between this indicator vector and the database DB. Specifically, this inner product is realized by computing the scalar multiplication of the generated Boolean share vector $Y_{j}^{\left(i\right)}\in {\left\{0,1\right\}}^{F_{\mathrm{maxlevel}}\left(t,p\right)}$ with each integer record DB[i], and accumulating the results via bitwise XOR to form the final response vector ${\mathrm{RES}}_{j}$.
(3) **Fault-Tolerant Reconstruction (**PIR.Rec**).** Suppose the client only receives responses from a surviving subset of servers $P_{\mathrm{rec}}$ where $|P_{\mathrm{rec}}|=t$. The client executes the iterative reconstruction algorithm over the received response vectors ${\left\{{\mathrm{RES}}_{i}\right\}}_{i\in P_{\mathrm{rec}}}$. Through the deterministic substitution of conflict parties, the client circumvents the missing servers and extracts the target record DB[α].

### 5.2. Correctness via Linear Homomorphism

The correctness of the retrieval relies on the XOR linear homomorphism of our reconstruction algorithm ${\mathrm{Rec}}_{\mathrm{tofp}}$. Because the reconstruction function consists solely of linear XOR operations, it commutatively penetrates the summation performed by the servers. The client’s reconstruction can be expressed as:

(5) $$\begin{array}{cc} y & ={\mathrm{Rec}}_{\mathrm{tofp}}\left(t,p,P_{\mathrm{rec}},P_{\mathrm{sub}},{\left\{{\mathrm{RES}}_{j}\right\}}_{j\in P_{\mathrm{rec}}}\right)\\ \; & ={\mathrm{Rec}}_{\mathrm{tofp}}\left(t,p,P_{\mathrm{rec}},P_{\mathrm{sub}},{\left\{\overset{N}{\underset{i=1}{⨁}}\left(Y_{j}^{\left(i\right)}\cdot \mathrm{DB}\left[i\right]\right)\right\}}_{j\in P_{\mathrm{rec}}}\right)\\ \; & =\overset{N}{\underset{i=1}{⨁}}\left[{\mathrm{Rec}}_{\mathrm{tofp}}\left(t,p,P_{\mathrm{rec}},P_{\mathrm{sub}},{\left\{Y_{j}^{\left(i\right)}\right\}}_{j\in P_{\mathrm{rec}}}\right)\right]\cdot \mathrm{DB}\left[i\right]. \end{array}$$

By the correctness of the underlying (t,p)-DPF, the evaluation ${\mathrm{Rec}}_{\mathrm{tofp}}$ yields the scalar 1 if and only if i=α, and 0 otherwise. Consequently, all non-target integer records are entirely canceled out, collapsing the equation exclusively to the desired term y=1·DB[α]=DB[α].

## 6. Security and Availability Analysis

In this section, we rigorously formalize our composite security paradigm and present the theoretical proofs demonstrating the privacy and high availability of the proposed (t,p)-FT-PIR scheme.

### 6.1. Security Paradigm and Assumptions

Recall the threat model formulated in Section 3.2; our architecture integrates diverse cryptographic primitives, yielding a composite security paradigm bound by the weakest link principle:

- The optimized RSS provides *perfect (information-theoretic) security* [22,27].
- The Randomized Special Combinatorial Design guarantees statistical security [20]. Specifically, for any unauthorized coalition of up to t−1 servers, the probability distributions of the seed assignments derived from $G_{0}$ and $G_{1}$ are statistically indistinguishable. We directly inherit the formal definitions and rigorous proofs of this statistical proximity from [20].
- The core DPF key expansion relies on Pseudorandom Generators (PRGs), inherently offering *computational security* [19].

Consequently, the overall protocol achieves *computational security* bounded by the underlying PRG against PPT adversaries. Furthermore, our scheme operates in the *plain model*, requiring no trusted setup or hybrid public-key infrastructure. We adopt a standard *simulation-based proof framework* [28] to demonstrate that the joint view of any unauthorized coalition can be perfectly simulated without any knowledge of the target index α.

### 6.2. Formal Privacy Proof

Given the established paradigm, we formally define the privacy requirement and provide the theoretical proof.

**Definition 5** ((t−1)-Computational Query Privacy)**.**
*Let λ be the security parameter and N be the database size. A (t,p)-FT-PIR protocol achieves (t−1)-computational query privacy if, for any target index α∈[N] and any unauthorized coalition of servers I⊂[p] bounded by |I|<t, there exists a PPT simulator Sim such that the joint view of the coalition is computationally indistinguishable from a simulated distribution:*

$$\left\{{\mathrm{View}}_{I}^{\mathrm{PIR}.\mathrm{Query}}\left(1^{\lambda },N,\alpha \right)\right\}\approx_{c}\left\{\mathrm{Sim}(1^{\lambda },N,I)\right\}$$

*where $\approx_{c}$ denotes computational indistinguishability, and ${\mathrm{View}}_{I}$ encompasses the generated query shares ${\left\{K_{i}\right\}}_{i\in I}$.*

**Theorem 1.** 
*Assuming the existence of secure PRGs, the proposed (t,p)-FT-PIR protocol achieves (t−1)-computational query privacy in the presence of static, semi-honest adversaries.*

**Proof.**  To prove Theorem 1, we construct a PPT simulator Sim and employ a standard hybrid argument [28] to establish indistinguishability between the real execution view and the simulated output.

**Simulator Construction:** The simulator Sim takes as input the security parameter λ, database capacity *N*, and the corrupted index set I (where |I|<t). Lacking the requisite threshold to reconstruct any valid tree path, Sim generates entirely independent and uniformly random bit-strings for all elements within the simulated query shares ${\left\{K_{i}\right\}}_{i\in I}$, expanding to the depth bounded by $F_{\mathrm{maxlevel}}\left(t,p\right)$. This simulation is executed independently of the real target index α.

**Hybrid Arguments:** We define a sequence of indistinguishable games, transitioning from the real protocol to the ideal simulation:

- **Game 0 (Real Protocol):** This represents the real execution of the PIR.Query algorithm. The query keys are deterministically expanded along the target path utilizing the authentic PRG, Combinatorial Design, and RSS configurations.
- **Game 1 (Computational Hybrid):** We modify Game 0 by substituting all PRG evaluations with a true uniformly random function. If a PPT adversary can distinguish Game 1 from Game 0, the adversary can be reduced to a distinguisher that breaks the underlying PRG assumption. Assuming computationally secure PRGs, Game 1 and Game 0 are computationally indistinguishable ($\approx_{c}$).
- **Game 2 (Statistical Hybrid):** Building upon Game 1, we replace the values derived from the Randomized Special Combinatorial Design with their ideal statistical distributions. Based on the inherent statistical security of the combinatorial design, the statistical distance between the real outputs and the ideal distribution is a negligible function in λ. Thus, Game 2 is statistically indistinguishable from Game 1 ($\approx_{s}$).
- **Game 3 (Information-Theoretic Hybrid):** Finally, we replace the RSS key shares allocated to the coalition I with purely independent and uniformly random shares. Because the optimized RSS framework guarantees perfect privacy for any subset smaller than the threshold *t*, the distribution of these shares is perfectly identical to uniformly random variables. Therefore, Game 3 is perfectly indistinguishable from Game 2 (≡).

**Conclusion:** Game 3 mathematically represents the exact behavior of our simulator Sim, outputting purely random strings for the coalition I. Because $\mathrm{Game}\;0\approx_{c}\mathrm{Game}\;1\approx_{s}\mathrm{Game}\;2\equiv \mathrm{Game}\;3$, we conclude that the real view is computationally indistinguishable from the simulated view. The (t−1)-computational query privacy is thus formally proven. □

### 6.3. Availability and Fault-Tolerance Analysis

**Theorem 2** (p−t Crash Fault Tolerance)**.**
*The proposed (t,p)-FT-PIR scheme deterministically guarantees the correct reconstruction of the target database record DB[α] provided that at least t servers remain responsive during the PIR.Eval phase.*

**Proof.**  Let $P_{\mathrm{rec}}\subseteq \left[p\right]$ denote the subset of surviving servers, where $|P_{\mathrm{rec}}|\geq t$. Without loss of generality, assume the client processes exactly *t* responses. Following the dimensional degradation mechanics of our generalized (t,p)-DPF defined previously, the iterative reconstruction algorithm ${\mathrm{Rec}}_{\mathrm{tofp}}$ dynamically resolves structural conflicts through deterministic delegation and conflict party substitution.

Because the recovery function relies exclusively on commutative XOR linear homomorphism, the summation penetrated by the surviving servers intrinsically isolates the target index. Specifically, the aggregated response matrices ${\left\{{\mathrm{RES}}_{i}\right\}}_{i\in P_{\mathrm{rec}}}$ naturally cancel out all non-target evaluations through parity decoding, collapsing exclusively to 1⊕DB[α]. Hence, the correctness mathematically holds with probability 1 as long as the threshold *t* is satisfied, circumventing up to p−t crash faults. □

## 7. Efficiency Evaluation

### 7.1. Theoretical Analysis

Let *N* denote the database size, *p* the total number of servers, *t* the reconstruction threshold, *q* the bit-length of each database record, and λ the security parameter. The maximum recursive sharing level is bounded by the function $F_{\mathrm{maxlevel}}\left(t,p\right)$ defined in Equation (4). Similar to [10,11,21], to bridge the gap between precise cryptographic implementation and macroscopic asymptotic evaluation, we first formulate the exact parameter-dependent complexities, followed by their asymptotic abstractions wherein p,t, and λ are treated as system constants.

**Computational complexity:**

- *Client side (PIR.Query and PIR.Rec)*: During the *query generation*, the hierarchical recursive patching mechanism generates at most $F_{\mathrm{maxlevel}}\left(t,p\right)$ components per server. Each component is derived from a base *t*-party DPF involving $O(t^{4}\cdot \mathrm{poly}\left(\lambda \right)\cdot \sqrt{N})$ PRG evaluations. Thus, the exact query generation complexity is $O(F_{\mathrm{maxlevel}}\left(t,p\right)\cdot t^{4}\cdot \mathrm{poly}\left(\lambda \right)\cdot \sqrt{N})$ symmetric operations. During *reconstruction*, recovering the target record requires lightweight bitwise XOR aggregations over the *t* surviving response vectors. This bounds the reconstruction overhead to O(t·q) bit operations, avoiding heavy algebraic interpolations entirely. By treating the threshold and security parameter as constants, the client’s overall asymptotic computation scales efficiently as $O(\sqrt{N})$.
- *Server side (PIR.Eval)*: Each server linearly scans the *N* database records. For each record, the server evaluates its local key set containing up to $F_{\mathrm{maxlevel}}\left(t,p\right)$ padded base keys, each requiring O(poly(t)) symmetric operations. Cumulatively, evaluating the entire database yields a deterministic exact computational complexity of $O(N\cdot F_{\mathrm{maxlevel}}\left(t,p\right)\cdot \mathrm{poly}\left(t\right)\cdot q)$ bit operations.

**Communication complexity:**

- *Request communication*: The query size per server corresponds to the local key set containing up to $F_{\mathrm{maxlevel}}\left(t,p\right)$ base DPF keys. Since a base *t*-party DPF key requires $O(t^{4}\cdot \mathrm{poly}\left(\lambda \right)\cdot \sqrt{N}+\sqrt{N})$ bits to represent the PRG seeds and the correction word cw, the exact request communication overhead scales as $O(F_{\mathrm{maxlevel}}\left(t,p\right)\cdot \left(t^{4}\cdot \mathrm{poly}\left(\lambda \right)\cdot \sqrt{N}+\sqrt{N}\right))$ bits per server. By normalizing p,t, and λ to O(1), this simplifies to an asymptotic request complexity of $O(\sqrt{N})$ elements.
- *Response communication*: The aggregated response vector ${\mathrm{RES}}_{j}$ inherits the dimension of the evaluated key, which is precisely bounded by $F_{\mathrm{maxlevel}}\left(t,p\right)$. Therefore, the exact response communication complexity from each server is entirely decoupled from the database size *N*, yielding a bound of $O(F_{\mathrm{maxlevel}}\left(t,p\right)\cdot q)$ bits. When measured in units of database elements, this overhead is determined by the threshold configuration $F_{\mathrm{maxlevel}}\left(t,p\right)$, entirely independent of *N*.

**Storage complexity:**

- *Client side*: The client operates in a purely stateless manner. Beyond allocating transient memory to execute queries and extract the reconstructed target DB[α], it maintains no persistent state, hints, or caching dictionaries across consecutive retrievals. This yields an optimal O(1) persistent client storage overhead with respect to *N*.
- *Server side*: In addition to permanently hosting the replicated database DB (which requires O(N·q) space), each server temporarily caches its assigned query key set during the PIR evaluation phase. Consequently, the auxiliary server-side storage overhead matches the exact request communication size. Specifically, our PIR scheme employs a (t,p)-DPF, which hierarchically relies on generating base (t,t)-DPF instances. The generation of each (t,t)-DPF requires $O(t^{4}\cdot \mathrm{poly}\left(\lambda \right))$ bits per row, originating from the cryptographic seeds generated in the initial phase ($G_{0}$). As visually supported by Figure 7, this structure scales over $\sqrt{N}$ rows, yielding a base footprint of $O(t^{4}\cdot \mathrm{poly}\left(\lambda \right)\cdot \sqrt{N})$. Factoring in the recursive accumulation across reduction levels from (t,p) down to (t,t), which is bounded by $F_{\mathrm{maxlevel}}\left(t,p\right)$, the total auxiliary server-side storage overhead is rigorously bounded by $O(F_{\mathrm{maxlevel}}\left(t,p\right)\cdot t^{4}\cdot \mathrm{poly}\left(\lambda \right)\cdot \sqrt{N})$ bits.

### 7.2. Experimental Analysis

**Implementation and Hardware.** We implement all protocols in C++ 23 using g++ 13.4.0 and a custom DPF over $Z_{2}$. Evaluations are driven by synthetic datasets (e.g., uniformly distributed) and randomly generated queries. All benchmarks are performed on an Ubuntu 22.04.5 LTS system (Intel Core i5-11300H @ 3.10 GHz, 12 GB RAM). Reported metrics represent the average across 100 independent iterations to minimize variance.

**Performance Evaluation of the Proposed Scheme**

To comprehensively evaluate the practical performance of the system, we first isolated the impact of system parameters (threshold *t*, server count *p*, and database scale *N*) on the query generation (PIR.Query), server evaluation (PIR.Eval), and client reconstruction (PIR.Rec) phases using a control variable approach under baseline configurations (fixed database item size of 256B).

**Figure 10:**
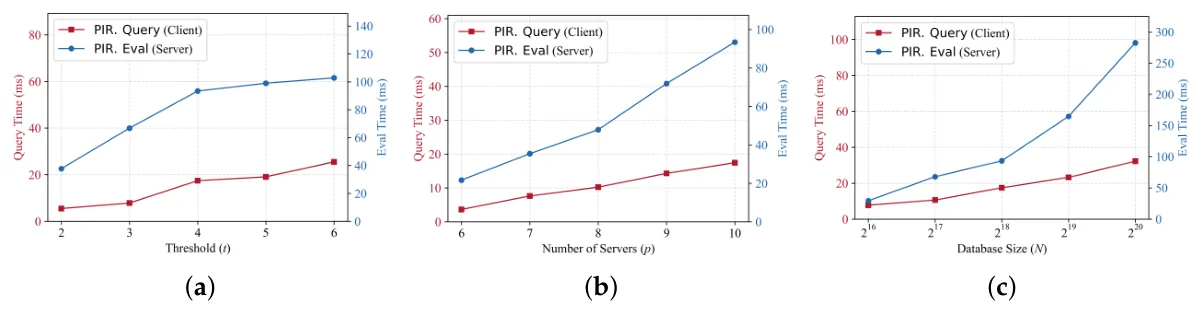
Query generation and server evaluation latency under varying parameters for a fixed item size of 256B. (**a**) Latency for varying threshold (*t*) with fixed $p=10,\;N=2^{18}$; (**b**) Latency for varying server count (*p*) with fixed $t=4,\;N=2^{18}$; (**c**) Latency for varying database size (*N*) with fixed t=4,p=10.

**Figure 11:**
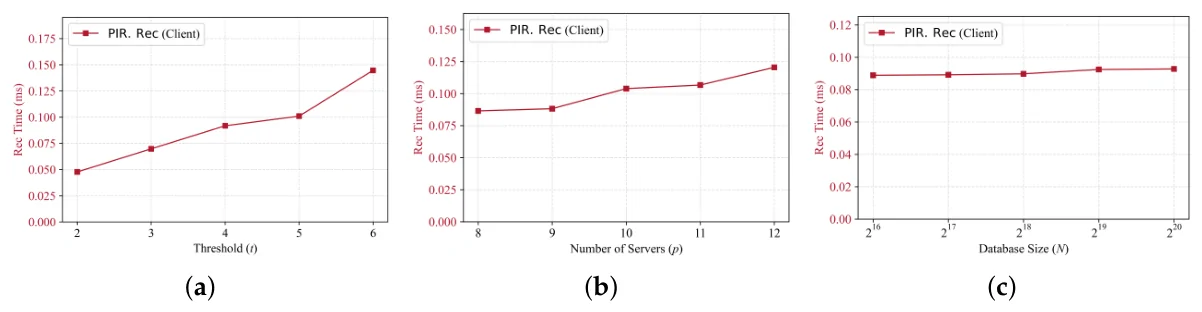
Client reconstruction latency under varying parameters for a fixed item size of 256B. (**a**) Latency for varying threshold (*t*) with fixed $p=10,\;N=2^{18}$; (**b**) Latency for varying server count (*p*) with fixed $t=4,\;N=2^{18}$; (**c**) Latency for varying database size (*N*) with fixed t=4,p=10.

- **Impact of Threshold (**t**):** As illustrated in Figure 10a and Figure 11a, with a fixed p=10 and $N=2^{18}$, both query generation and server evaluation latencies exhibit a controlled proportional growth as the threshold *t* increases from 2 to 6. Algorithmically, this is because a larger *t* requires the hierarchical recursive patching mechanism to generate more foundational DPF key blocks to resolve higher-order access conflicts, which consequently increases the number of pseudorandom generator (PRG) invocations and local vector computations on the server side. Meanwhile, although the reconstruction time also shows a slight upward trend with *t*, its peak remains at an extremely low ∼0.19ms. This is because our reconstruction process completely abandons heavy algebraic interpolations, relying instead on deterministic response substitutions and straightforward XOR aggregations; the increase in *t* merely adds a linear stack of XOR operations, thereby preserving its exceptionally lightweight nature.
- **Impact of Server Count (**p**):** As shown in Figure 10b and Figure 11b, with a fixed t=4 and $N=2^{18}$, the query and evaluation times steadily increase as the server cluster scale *p* expands from 6 to 10. This is due to the fact that for every newly introduced node, the distribution algorithm must allocate new conflict-resolution subkeys and copy states, thereby increasing the overall generation and traversal workload across the network. However, the client reconstruction time exhibits minimal sensitivity to changes in *p* (consistently remaining below 0.13ms), demonstrating that this mechanism does not impose additional computational pressure on the receiver when scaling the cluster.
- **Impact of Database Size (**N**):** As illustrated in Figure 10c and Figure 11c, with a fixed t=4 and p=10, the server evaluation time grows linearly with the database size *N*, successfully completing heavy-load benchmark tests for massive data up to the $2^{20}$ scale. The core reason is that the evaluation phase requires a comprehensive scalar-vector multiplication traversal over the entire database. In stark contrast, the client reconstruction time forms a perfect horizontal line at approximately 0.10ms, completely decoupled from the database size *N*. This phenomenon algorithmically confirms that the server side has precisely compressed the target data into a fixed-dimension response vector via the XOR linear homomorphism mechanism, allowing the client to simply perform computations on the aggregated results, completely bypassing the computational bottleneck associated with data volume expansion.

**Comparison with Existing Scheme**

To further highlight the architectural advantages of our design, we rigorously compared our scheme with the state-of-the-art fault-tolerant PIR scheme [21].

**Figure 12:**
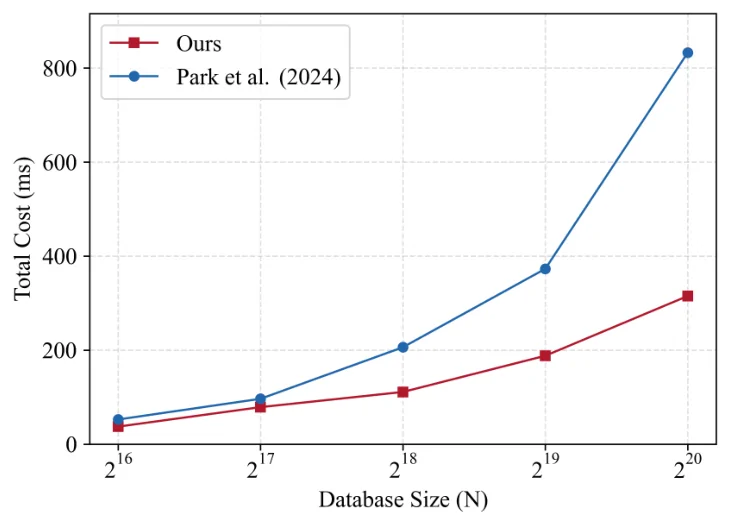
Total end-to-end latency comparison across different database sizes for a fixed item size of 256B, p=10, and t=4, including the scheme of Park et al. [21].

- **Computation Overhead and End-to-End Latency:** Figure 12 presents the total end-to-end latency of the system across varying database scales *N* under a fixed item size of 256B, p=10, and t=4. Across all evaluated data scales, the total latency of our scheme significantly outperforms scheme [21]. At the maximum tested scale of $N=2^{20}$, our protocol takes approximately 300ms in total, whereas scheme [21] exceeds 800ms. This substantial performance lead is primarily attributed to fundamental differences in computational paradigms: scheme [21] relies on a Shamir-based threshold mechanism, forcing the client to perform heavy polynomial interpolations during reconstruction. In contrast, our scheme replaces complex algebraic operations with ultra-low-overhead linear XOR aggregation, maintaining low latency even under heavy workloads.
- **Empirical Communication Overhead:** To accurately capture real-world network utilization, we strictly measured the actual serialized communication costs at the underlying layer during system execution. Table 3 details the upload (Request) and download (Response) communication costs across different database scales. On the request side, our scheme incurs a slightly larger overhead. This is because achieving robustness against single-point failures requires transmitting a large number of polynomial sharing components generated by the hierarchical expansion to the servers. However, during the download response phase, our scheme demonstrates a significant advantage: the response size remains constant at 4.25KB, independent of *N*. In contrast, the response volume of scheme [21] inflates aggressively as the database grows (surpassing 1281KB at $N=2^{20}$). The fundamental reason for this stark contrast is that scheme [21] must force servers to return multi-level verification blocks tied to the data scale to filter out failed nodes; our design, however, offsets failure paths through implicit parity checks during the local evaluation phase itself. This design completely eliminates the downstream network congestion bottleneck at the expense of a fraction of the relatively abundant uplink bandwidth, indicating its suitability for large-scale databases.

## 8. Related Work

### 8.1. Single-Server PIR

Single-server PIR fundamentally operates under the computational privacy setting. The existing literature typically optimizes its performance bottleneck through two distinct technical routes: relying on homomorphic encryption to compress communication, or utilizing hint-based preprocessing to bypass heavy public-key computations.

#### 8.1.1. HE-Based Schemes

Scheme [8] proposed an unkeyed doubly efficient PIR (DEPIR) protocol based on the standard ring-LWE assumption, establishing a robust theoretical foundation for sublinear online query complexity. However, extending this construction to RAM-FHE necessitates an additional circular security assumption, and the protocol inherently incurs prohibitive ciphertext storage overhead. To mitigate the offline communication bottleneck and enhance server throughput, scheme [6] introduced “silent preprocessing” via LWE-to-RLWE homomorphic packing techniques and number theoretic transform (NTT) acceleration. Nevertheless, to successfully compress the downstream response, this approach inevitably inflates the client’s online query size. To address the circuit depth and computational bottlenecks encountered during the preprocessing phase, scheme [7] utilized homomorphic Thorp permutations combined with a learning with rounding (LWR)-based PRG to construct a constant-depth preprocessing circuit. Although this architecture reduces offline processing time and achieves sublinear offline bandwidth, it imposes a bound on the maximum number of queries and fails to eliminate the substantial constant-factor overhead inherent in homomorphic evaluations. Consequently, the prohibitive cryptographic computational costs remain the primary impediment to the practical deployment of HE-based PIR frameworks.

#### 8.1.2. Hint-Based Preprocessing Schemes

Distinct from HE-based approaches, another line of research leverages lightweight symmetric primitives via client-side preprocessing. Scheme [9] introduced puncturable pseudorandom sets to achieve sublinear online communication and computation under standard one-way function assumptions. Building on this, schemes [10,11] proposed hint-based constructions using pseudorandom functions and programmable pseudorandom sets, systematically reducing the online communication complexity to $O(\sqrt{N})$ and $O(N^{1/4})$, respectively. Furthermore, to optimize the parameter configurations for stateful clients, scheme [12] designed a dynamic data relocation mechanism. This approach precisely balances the client’s local storage overhead (*S*) and the server’s online computation time (*T*), achieving a space–time trade-off of S·T=O(N). While these preprocessing protocols significantly improve online efficiency without relying on public-key operations, they fundamentally require an O(N) offline database scan to fetch hints. Additionally, the bounded nature of these pre-fetched hints necessitates costly full-scale resets upon depletion, which limits their applicability in continuous high-frequency retrieval scenarios.

### 8.2. Multi-Server PIR

To bypass the inherent computational and storage bottlenecks of single-server models, multi-server PIR architectures distribute queries across multiple nodes. By amortizing the workload, this architecture significantly improves retrieval efficiency. Existing multi-server protocols primarily evolve along two distinct technical trajectories: information-theoretic constructions based on algebraic coding, and lightweight cryptographic protocols utilizing FSS.

#### 8.2.1. Algebraic and Coding-Based Schemes

Operating under the information-theoretic privacy setting, these schemes leverage polynomials and error-correcting codes to mask client queries against bounded server collusion [13]. Early foundational works established baselines: scheme [14] bounded 2-server communication at $O(N^{1/3})$, while scheme [15] achieved optimal Byzantine robustness via algebraic decoding algorithms. Subsequent research successfully breached these initial limits. To optimize bandwidth, scheme [16] utilized matching vector codes to compress communication to a sub-polynomial level. Furthermore, to mitigate the O(N) server computation barrier, scheme [17] proposed a multi-server doubly efficient PIR paradigm, employing preprocessing to amortize online computation to a sublinear level. Despite these advancements, such schemes rely heavily on expensive algebraic operations over large finite fields and stateful preprocessing mechanisms. Consequently, they remain fundamentally constrained in practical deployments, struggling to simultaneously deliver logarithmic communication, stateless client operations, and efficient fault tolerance.

#### 8.2.2. FSS-Based Schemes

Traditional PIR schemes typically incur prohibitively high communication overhead. To alleviate this bottleneck, schemes [18,19] introduced the DPF and FSS frameworks. By transmitting key shares generated via pseudorandom generators (PRGs), these primitives compress the client’s upload communication overhead from linear to sublinear. Building upon this foundation, subsequent work [19] further optimized the tree-based key expansion mechanism in the two-server architecture, bounding the request communication complexity to O(logN) while maintaining a response communication of O(1) with respect to *N*. Such constructions achieve asymptotically optimal communication in the considered two-server setting. However, their applicability is restricted to the two-server model, rendering them vulnerable to more sophisticated collusion attacks.

To accommodate distributed environments with heightened security requirements, researchers have endeavored to extend FSS to multi-party (p≥3) architectures. However, early multi-party DPF protocols relying on deterministic constructions suffered from severe bandwidth inflation; their request overhead reached $O(\sqrt{N}\cdot 2^{p})$, which grows exponentially with the number of servers [18]. To circumvent this theoretical barrier, scheme [20] proposed an optimization scheme based on a randomized special combinatorial design, successfully compressing the key size of multi-party DPFs to $O(p^{3}\sqrt{N})$. Despite this significant improvement in query efficiency, standard FSS-based PIR protocols heavily rely on the responses from all *p* servers during the reconstruction phase. Consequently, a single point of failure or network latency can result in the complete failure of the retrieval process. To enhance system availability, scheme [21] introduced a fault-tolerant PIR protocol that allows the client to recover the target data using only a subset of responses from active nodes. However, its underlying mechanism necessitates returning multiple verification blocks to the client during the evaluation phase, which severely erodes the inherent O(1) lightweight response advantage of standard DPF protocols.

## 9. Conclusions and Future Work

This paper presents a novel FT-PIR protocol that resolves single-point failure vulnerabilities in standard FSS-based architectures. Built on a new FT-DPF with a hierarchical recursive patching mechanism, our scheme enables deterministic reconstruction from any *t* surviving servers. Crucially, it completely decouples the response overhead from the database size *N*, achieving a $O(F_{\mathrm{maxlevel}}\left(t,p\right))$ communication bound. By replacing expensive algebraic interpolations with lightweight XOR aggregations, the protocol ensures highly efficient, stateless client-side reconstruction. Ultimately, it provides robust fail-stop fault tolerance while guaranteeing (t−1)-computational privacy under the semi-honest model.

Future work will focus on two extensions: (1) achieving full Byzantine fault tolerance against malicious adversaries, and (2) adapting our recursive patching mechanism to expressive FSS primitives, such as distributed comparison functions (DCFs), to support privacy-preserving range queries and complex analytics.

## Figures and Tables

**Table 1:** Comparison with prior works.

Schemes	Fault-Tolerant	Offline Communication	Request Communication	Response Communication	Extra Space
[10]	×	O(N)	$O(\sqrt{N})$	O(1)	$O(\sqrt{N})$
[11]	×	O(N)	$O(N^{1/4})$	$O(N^{1/4})$	$O(\sqrt{N})$
[18]	×	0	$O(2^{p}\cdot \sqrt{N})$	O(1)	O(1)
[21]	✓	0	$O(\sqrt{N})$	$O(\sqrt{N})$	O(1)
Ours	✓	0	$O(\sqrt{N})$	$O(F_{\mathrm{maxlevel}}\left(t,p\right)$)	O(1)

Here, *N* denotes the number of items in the database, *p* denotes the number of servers, *t* denotes the threshold. $F_{\mathrm{maxlevel}}\left(t,p\right)$ denotes the maximum sharing level function as defined in Equation (4). For clarity in asymptotic comparison, the communication complexities are measured in terms of the number of database records, implicitly normalizing the record size q,t and security parameter λ to O(1). The extra space denotes the client’s extra storage.

**Table 2:** Summary of core notations.

Notation	Meaning
λ	Security parameter.
[p]	The set of integers {1,2,…,p}.
N,u	PIR database size and its square-root matrix dimension ($u=\sqrt{N}$).
α	The target query index in the PIR database (α∈[N]).
p,t	Total number of servers and the reconstruction threshold.
ℓ,r	Number of base PRG seeds *ℓ* and additional planted seeds *r*.
$S_{i}^{g}$	Set of PRG seeds allocated to server *i* for row *g*.
PRG	Pseudorandom generator $\mathrm{PRG}:{\left\{0,1\right\}}^{\lambda }\to {\left\{0,1\right\}}^{u}$.
$e_{\delta }$	*u*-dimensional unit vector with 1 at the δ-th position and 0 elsewhere.
cw	*u*-dimensional correction word hiding the column coordinate δ for parity-based noise cancellation.
conflict	The conflict party index ($i-2^{\lceil \log_{2}i\rceil -1}$ for t=2, i−t for t>2) for resolving key structural conflicts.
$F_{\mathrm{maxlevel}}\left(t,p\right)$	Maximum sharing level function bounding the recursion depth and share expansion per server, detailed in Equation (4).
$P_{\mathrm{sub}}$	An ordered list of designated server indices in the current recursive level.
$P_{\mathrm{rec}}$	The subset of active servers ($|P_{\mathrm{rec}}|=t$) surviving and participating in the reconstruction.
Ind	The 1-based index mapping array for the elements in $P_{\mathrm{rec}}$ relative to $P_{\mathrm{sub}}$.
reclevel	The target active level (depth) tracked during iterative conflict resolution.
$K_{j}$	The DPF evaluation key set (query share) allocated to server *j*.
*q*	Bit-length of each database record DB[i] (i.e., $\mathrm{DB}\left[i\right]\in {\left\{0,1\right\}}^{q}$).
${\mathrm{RES}}_{j}$	The local evaluation result (aggregated response vector) returned by server *j*.
*y*	The reconstructed output value.

**Table 3:** Communication cost comparison under varying database sizes *N* for fixed item size 256B, p=10, and t=4 (unit: KB).

Database Size *N*	Ours		[21]	
	Request	Response	Request	Response
$2^{16}$	940.71	4.25	40.04	321.29
$2^{17}$	1320.52	4.25	56.60	453.79
$2^{18}$	1864.32	4.25	80.04	641.29
$2^{19}$	2645.60	4.25	113.32	907.54
$2^{20}$	3705.10	4.25	160.04	1281.29

## Data Availability

The original contributions presented in this study are included in the article. Further inquiries can be directed to the corresponding author.
